# Design, Synthesis and Antimycobacterial Activity of Novel Imidazo[1,2-*a*]pyridine Amide-Cinnamamide Hybrids

**DOI:** 10.3390/molecules21010049

**Published:** 2015-12-30

**Authors:** Linhu Li, Zhuorong Li, Mingliang Liu, Weiyi Shen, Bin Wang, Huiyuan Guo, Yu Lu

**Affiliations:** 1 Institute of Medicinal Biotechnology, Chinese Academy of Medical Sciences and Peking Union Medical College, Beijing 100050, China; lilinhu0627@163.com (L.L.); l-z-r@263.net (Z.L.); guohuiyuan108@126.com (H.G.); 2 Zhejiang Starry Pharmaceutical Co. Ltd., Xianju 317300, China; swy109@starrypharma.com; 3 Beijing Key Laboratory of Drug Resistance Tuberculosis Research, Department of Pharmacology, Beijing Tuberculosis and Thoracic Tumor Research Institute, Beijing Chest Hospital, Capital Medical University, Beijing 101149, China; chaofanguodu123@163.com

**Keywords:** imidazo[1,2-*a*]pyridine amide-cinnamamide hybrids, design, synthesis, antimycobacterial activity

## Abstract

We report herein the design and synthesis of a series of novel imidazo[1,2-*a*]pyridine amide-cinnamamide hybrids linked via an alkyl carbon chain. All 38 new hybrids were evaluated for their antimycobacterial activity against *M. tuberculosis* (MTB) H37Rv ATCC 27294 using the microplate Alamar Blue assay (MABA). Although the hybrids are less active than the two reference compounds, the promising activity (MICs: 4 μg/mL) of 2,6-dimethylimidazo[1,2-*a*]pyridine amide-cinnamamide hybrids **11e** and **11k** could be a good starting point to further find new lead compounds against multi-drug-resistant tuberculosis.

## 1. Introduction

Tuberculosis (TB), including multi-drug-resistant TB (MDR-TB) and extensively-drug-resistant TB (XDR-TB), as well as the lethal combination represented by HIV co-infection, constitutes an unacceptable burden of human suffering and loss [1,2]. For example, the current therapy requires at least 20 months of treatment for MDR-TB. For these infections, several novel candidates are currently in clinical trials [3,4,5], and one of them, Bedaquiline, was approved by the FDA in December 2012 for the treatment of MDR-TB. However, its wide application may be limited because of serious adverse effects, such as cardiac arrhythmias [6]. Therefore, there is still an urgent need for new anti-TB drugs that target novel biological pathways in *M. tuberculosis* (MTB), shorten therapy and reduce the burden of latent infection [7].

**Figure 1:**
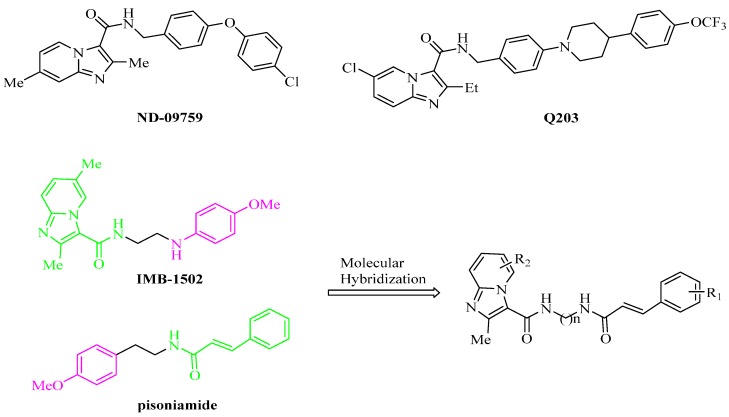
Structures of selected anti-tuberculosis (TB) compounds and the design of imidazo[1,2-*a*]pyridine amide-cinnamamide hybrids.

In recent years, imidazo[1,2-*a*]pyridine amides (IPAs) targeting the QcrB subunit of the menaquinol cytochrome c oxidoreductase (bc1 complex), which is a critical component of mycobacterial energy metabolism [8], have attracted broad attention due to their potent activity against MTB-resistant and -sensitive strains [9,10,11,12,13,14,15]. Two promising drug candidates, ND-09759 (Figure 1) and Q203 (Figure 1), are currently in pre-clinical and phase I clinical development [11,16], respectively. It has been generally accepted that the carboxamide linker with the *N*-benzylic group is critical for anti-MTB activity [12], but IPA derivatives containing a *N*-(2-phenoxyl)ethyl or *N*-(2-phenylamino)ethyl moiety, such as IMB1502 (Figure 1), were observed to have nanomolar potency against MTB H37Rv and MDR-MTB strains in our lab [17].

On the other hand, *trans*-cinnamic acid derivatives are an important class of molecules by reason of their wide spectrum of pharmacological profiles, including antioxidative [18], antitumor [19], antibacterial [20] and antitubercular [21] properties. It is of interest to note that cinnamic acid was used for TB even before the current therapy was discovered [22]. Additionally, cinnamic acid was found to act synergistically with isoniazid, rifamycin and other known anti-TB agents against MTB [23]. Additionally, rifamycin SV, a hybrid derivative of cinnamic acid and rifamycin, was observed to show higher activity against most of the tested MTB and MDR-MTB strains than its individual counterparts [24]. Recently, several natural products containing a cinnamic acid moiety were reported as anti-TB agents [25,26,27,28], such as pisoniamide (Figure 1), a natural cinnamamide isolated from *Pisonia aculeate* [29].

In our continuous program in the search of potent and safe IMB1502 derivatives, we intended to construct a new class of hybrids as attractive anti-TB agents by molecular hybridization between IMB1502 and pisoniamide. A detailed structural comparison revealed that both of them are composed of a delocalized aromatic pharmacophore (green) and a hydrophobic moiety (purple) connected via a same ethylidyne linkage (black, Figure 1). Therefore, a series of novel hybrid structures containing IPA and cinnamamide moieties linked via an alkyl carbon chain (ethylidyne or propylidyne) were designed and synthesized in this study (Figure 1), with the hope that these target compounds would exhibit improved anti-MTB activity.

## 2. Results and Discussion

### 2.1. Chemistry

**Scheme 1:**
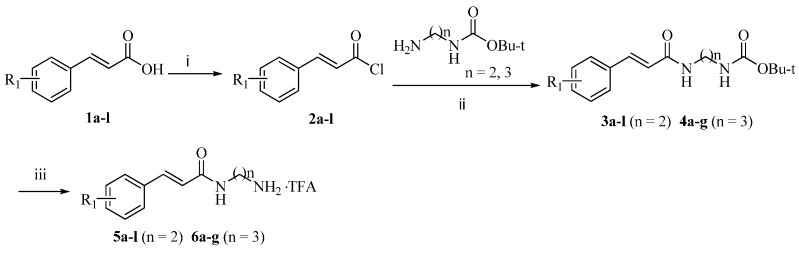
Synthesis of cinnamamide derivatives **5a**–**l** and **6a**–**g**. *Reagents and conditions*: (i) SOCl_2_, DMF, relux, 4 h; (ii) Net_3_, CH_2_Cl_2_, rt, 2 h, 59%–75% (for two steps); (iii) TFA CHCl_3_, rt, 1 h, 100%.

**Scheme 2:**
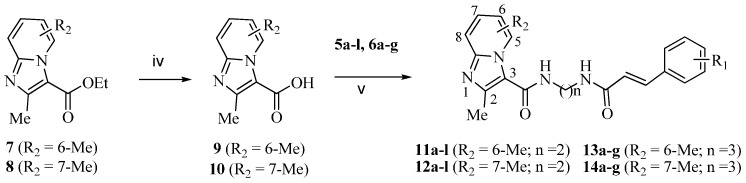
Synthesis of imidazo[1,2-*a*]pyridine amide-cinnamamide hybrids **11**–**14**. *Reagents and conditions*: (iv) LiOH, EtOH, rt, overnight, 78%–87%; (v) BOP-Cl, Net3, CH2Cl2, rt, 2 h, 32%–72%.

Detailed synthetic pathways to cinnamamide derivatives **5**–**6** and novel hybrids **11**–**14** are depicted in Scheme 1 and Scheme 2, respectively. Commercially available cinnamic acids **1a**–**l** were treated with thionyl chloride at reflux to give the corresponding acyl chlorides **2a**–**l**. Condensation of the resulting **2a**–**l** with tert-butyl (2-aminoethyl)carbamate or tert-butyl (3-aminopropyl)carbamate in the presence of triethylamine (NEt_3_) yielded **3a**–**l** and **4a**–**g**, respectively, which were hydrolyzed with trifluoroacetic acid (TFA) to afford the desired cinnamamide derivatives **5a**–**l** and **6a**–**g** as TFA salts (Scheme 1).

The target Compounds **11**–**14** were conveniently prepared from ethyl 2,6-dimethylimidazo[1,2-*a*]pyridine-3-carboxylate **7** and ethyl 2,7-dimethylimidazo[1,2-*a*]pyridine-3-carboxylate **8** [30,31], by hydrolysis in LiOH–EtOH and condensation with the above cinnamamide derivatives **5a**–**l** and **6a**–**g** in the presence of bis(2-oxo-3-oxazolidinyl)phosphonic chloride (BOP-Cl) and NEt_3_, successively (Scheme 2).

### 2.2. Anti-MTB Activity

The target Compounds **11**–**14** were evaluated for their *in vitro* activity against MTB H37Rv ATCC 27294 using the microplate Alamar Blue assay (MABA) [32,33]. The minimum inhibitory concentration (MIC) is defined as the lowest concentration effecting a reduction in fluorescence of ≥90% relative to the mean of replicate bacterium-only controls, and MICs of **11**–**14** along with IMB1502 and isoniazid (INH) for comparison are presented in Table 1. The data reveal that all of the new synthesized hybrids **11**–**14** (MICs: 4–>32 μg/mL) are much less active than the reference drug INH (MIC: 0.05 μg/mL) and the parent compound IMB1502 (MIC: 0.015 μg/mL), but fourteen of them have potential activity against this strain (MICs: 4–32 μg/mL). Among them, Compounds **11e** and **11k** display the highest activity (MICs: 4 μg/mL), and Compounds **11k** and **11e** have also promising activity with MICs of 8 and 16 μg/mL, respectively, against MTB H37Rv ATCC 27294.

Variations (R_1_) on the benzene ring of the cinnamamide moiety in this study include methyl, methoxy, trifluoromethyl, trifluoromethoxy, nitro, fluoro, chloro, 3,4-dichloro, 3,4,5-trimethoxyl and hydrogen substitution (Table 1). Generally, compounds with multi-substituents (3,4-dichloro, 3,4,5-trimethoxyl) or without a substituent (hydrogen) on the benzene ring, like many mono-substituted (fluoro, chloro, methyl, methoxy, nitro) hybrids, are inactive (MICs: ≥32 μg/mL) in this study. In the case of the hybrids with MICs of 4–16 μg/mL, the 2,6-dimethylimidazo[1,2-*a*]pyridineamide moiety is more active than the responding 2,7-dimethylimidazo[1,2-*a*]pyridineamide one (**11e** *vs.* **12e**; **11k** *vs.* **12k**). On the other hand, the hybrids with an ethylidyne linker (*n* = 2) seem to be more potent than the analogs containing a propylidyne one (*n* = 3) (**11e** *vs.* **13e**; **12e** *vs.* **14e**), which is consistent with the structure-activity relationship (SAR) in our previous study [17]. Moreover, in the series of Hybrids **11** and **12** (*n* = 2), introduction of an electron-donating mono-substituted group (R_1_ = methyl, methoxy) instead of an electron-withdrawing one (R_1_ = trifluoromethyl, trifluoromethoxy) is significantly detrimental to the activity (**11a** *vs.* **11e**; **11d** *vs.* **11k**; **12a** *vs.* **12e**; **12d** *vs.* **12k**). Finally, representative IMPs (**5e**, **6e**) and cinnamamide (**15**) were, as expected, found to have no anti-MTB activity (MICs: >32 μg/mL), which highlights the design rationality of our hybrids in this study.

**Table 1:** Anti-*M. tuberculosis* (MTB) activity of imidazo[1,2-*a*]pyridine amide-cinnamamide Hybrids **11**–**14**. 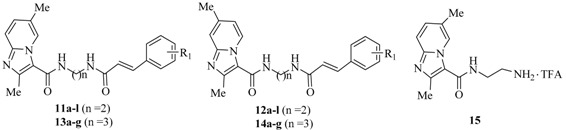

Compound	R_1_	MIC (μg/mL)	Compd.	R_1_	MIC (μg/mL)
**11a**	4-CH_3_	>32	**12a**	4-CH_3_	>32
**11b**	3,4,5-tri-OCH_3_	>32	**12b**	3,4,5-tri-OCH_3_	>32
**11c**	3,4-di-Cl	>32	**12c**	3,4-di-Cl	32
**11d**	4-OCH_3_	>32	**12d**	4-OCH_3_	>32
**11e**	4-CF_3_	4	**12e**	4-CF_3_	16
**11f**	2-F	>32	**12f**	2-F	>32
**11g**	4-F	>32	**12g**	4-F	>32
**11h**	3-F	>32	**12h**	3-F	>32
**11i**	4-Cl	>32	**12i**	4-Cl	>32
**11j**	4-NO_2_	32	**12j**	4-NO_2_	>32
**11k**	4-OCF_3_	4	**12k**	4-OCF_3_	8
**11l**	H	>32	**12l**	H	>32
**13a**	4-CH_3_	32	**14a**	4-CH_3_	>32
**13b**	3,4,5-tri-OCH_3_	32	**14b**	3,4,5-tri-OCH_3_	>32
**13c**	3,4-di-Cl	>32	**14c**	3,4-di-Cl	>32
**13d**	4-OCH_3_	>32	**14d**	4-OCH_3_	32
**13e**	4-CF_3_	32	**14e**	4-CF_3_	32
**13f**	2-F	32	**14f**	2-F	>32
**13g**	4-F	32	**14g**	4-F	32
**5e**		>32	isoniazid		0.05
**6e**		>32	IMB1502		0.015
**15**		>32			

## 3. Materials and Methods

### 3.1. Chemistry

Melting points were determined in open capillaries and are uncorrected. ^1^H-NMR spectra were determined on a Varian Mercury-400 spectrometer in DMSO-*d*_6_, D_2_O or CDCl_3_ using tetramethylsilane as an internal standard (see Supplementary Materials). Electrospray ionization (ESI) mass spectra and high resolution mass spectra (HRMS) were obtained on an MDSSCIEX Q-Tap mass spectrometer. The reagents were all of analytical grade or chemically pure. TLC was performed on silica gel plates (Merck, ART5554 60F254, Kenilworth, NJ, USA).

### 3.2. Synthesis

#### 3.2.1. General Procedure for the Synthesis of Imidazo[1,2-*a*]pyridine-3-carboxylic Acids **9**, **10**

To a solution of **7**, **8** (4.0 mmol) in EtOH (30 mL) was added an aqueous solution of lithium hydroxide (12.0 mmol in 10 mL of water), and the mixture was stirred at room temperature overnight. The organic solvent was evaporated, and 1N HCl was added until the pH = 6. The residual was collected by filtration, washed with water and dried to give **9**, **10**.

*Ethyl 2,6-dimethylimidazo[1,2-a]pyridine-3-carboxylate acid* (**9**)*:* The title compound was obtained from **7** as a white solid (87%); m.p.: 173–175 °C. ^1^H-NMR (500 MHz, DMSO-*d*_6_) δ (ppm): 12.93 (s, 1H, -COOH), 9.08 (s, 1H, pyridine-H), 7.55 (d, *J =* 9.1 Hz, 1H, pyridine-H), 7.36 (dd, *J* = 9.1, 1.6 Hz, 1H, pyridine-H), 2.57 (s, 3H, CH_3_), 2.35 (s, 3H, CH_3_). MS-ESI (*m*/*z*): 191 [M + H]^+^.

*Ethyl 2,7-dimethylimidazo[1,2-a]pyridine-3-carboxylate acid* (**10**)*:* The title compound was obtained from **8** as a white solid (78%); m.p.: 161–163 °C. ^1^H-NMR (400 MHz, DMSO-*d*_6_) δ (ppm): 12.90 (s, 1H, -COOH), 9.12 (d, *J* = 7.1 Hz, 1H, pyridine-H), 7.47–7.38 (m, 1H, pyridine-H), 6.98 (dd, *J* = 7.1, 1.7 Hz, 1H, pyridine-H), 2.56 (s, 3H, CH_3_), 2.40 (s, 3H, CH_3_). MS-ESI (*m*/*z*): 191 [M + H]^+^.

#### 3.2.2. General Procedure for the Synthesis of Cinnamamide Derivatives **5a**–**l** and **6a**–**g**

To a solution of 1.2 equiv of substituted cinnamic acid **1a**–**l** (5 mmol) in 5 equiv of thionyl chloride (3.6 mL), a catalytic amount of DMF was added. The reaction mixture was refluxed for 4 h, and then, solvent was evaporated under vacuum to get the product **2a**–**l** in the form of a solid residue in quantitative yield. The solid residue was directly added partially to an ice-cold stirred solution of 1.0 equiv of *tert*-butyl (2-aminoethyl)carbamate or tert-butyl (3-aminopropyl)carbamate and 2.0 equiv triethylamine in DCM (20 mL). After the addition, the mixture was warmed to room temperature and stirred for 2 h. Then, DCM (20 mL) was added and washed with 0.2 M HCl (40 mL), H_2_O (40 mL), 5% saturated. NaHCO3 (40 mL) and brine (40 mL), then dried over anhydrous magnesium sulfate. The solvent was removed *in vacuo* to give the corresponding cinnamamide derivatives **3a**–**l** (65%–75%, from **1a**–**l**) and **4a**–**g** (59%–70%, from **1a**–**g**) as a white solid. **3a**–**l**, **4a**–**g** (4 mmol) in DCM/TFA (9:1, 40 mL) were stirred at room temperature for 1 h. Solvents were removed *in vacuo* to yield **5a**–**l** (100%) and **6a**–**g** (100%) as a colorless oil.

#### 3.2.3. General Procedure for the Synthesis of Imidazo[1,2-*a*]pyridine amide-cinnamamide Hybrids **11**–**14**

A mixture of imidazo[1,2-*a*]pyridine-3-carboxylic acids **9**, **10** (1.0 mmol) and BOP-Cl (1.2 mmol) in dry DCM (10 mL) was stirred under N_2_ at room temperature for 5 min at room temperature. Then, Et_3_N (2.2 mmol) was added followed by **5a**–**l** and **6a**–**g** (1.2 mmol). The resulting suspension was allowed to continue stirring for 3 h. DCM (10 mL) was added and washed with 0.2 M HCl (20 mL), H_2_O (20 mL), 5% satd. NaHCO_3_ (20 mL) and brine (20 mL), then dried over anhydrous magnesium sulfate. The solvent was removed *in vacuo*. Silica flash column chromatography eluting with DCM/MeOH (95:5) yielded **11**–**14**.

*(E)-2,6-Dimethyl-N-(2-(3-p-tolylacrylamido)ethyl)imidazo[1,2-a]pyridine-3-carboxamide* (**11a**): The title compound was prepared from **5a** and **9** as a white solid (61%); m.p.: 215–217 °C. ^1^H-NMR (500 MHz, DMSO-*d*_6_) δ (ppm): 8.82 (s, 1H, pyridine-H), 8.27 (d, *J* = 5.5 Hz, 1H, -CONH-), 7.86 (d, *J* = 5.0 Hz, 1H, -CONH-), 7.47–7.44 (m, 3H, Ar-H), 7.40 (d, *J* = 16.0 Hz, 1H, =C-H), 7.23–7.20 (m, 3H, Ar-H), 6.58 (d, *J* = 16.0 Hz, 1H, =C-H), 3.44–3.41 (m, 4H, 2 × -CH_2_-), 2.54 (s, 3H, CH_3_), 2.31 (s, 3H, CH_3_), 2.28 (s, 3H, CH_3_). ^13^C-NMR (126 MHz, DMSO-*d*_6_) δ (ppm): 165.98, 161.57, 145.33, 144.25, 139.68, 139.14, 132.57, 130.00, 129.63, 127.96, 125.22, 122.39, 121.53, 116.14, 115.92, 38.96, 21.41, 18.24, 16.07. MS-ESI (*m*/*z*): 377 [M + H]^+^.

*(E)-2,6-Dimethyl-N-(2-(3-(3,4,5-trimethoxyphenyl)acrylamido)ethyl)imidazo[1,2-a]pyridine-3-carboxamide* (**11b**): The title compound was obtained from **5b** and **9** as a white solid (63%)’ m.p.: 224–226 °C. ^1^H-NMR (500 MHz, DMSO-*d*_6_) δ (ppm): 8.83 (s, 1H, pyridine-H), 8.24 (t, *J* = 5.0 Hz, 1H, -CONH-), 7.85 (t, *J* = 5.0 Hz, 1H, -CONH-), 7.47 (d, *J* = 9.0 Hz, 1H, pyridine-H), 7.38 (d, *J* = 15.5 Hz, 1H, =C-H), 7.23 (dd, *J* = 9.0, 1.5 Hz, 1H, pyridine-H), 6.90 (s, 2H, Ar-H), 6.60 (d, *J* = 15.5 Hz, 1H, =C-H), 3.80 (s, 6H, -OCH3), 3.67 (s, 3H, CH3), 3.44–3.41 (m, 4H, 2 × -CH2-), 2.55 (s, 3H, CH3), 2.28 (s, 3H, CH3). ^13^C-NMR (126 MHz, DMSO-*d*_6_) δ (ppm): 165.48, 161.13, 153.08, 144.90, 143.82, 138.93, 138.60, 130.50, 129.18, 124.78, 121.95, 121.45, 115.68, 115.49, 104.91, 60.10, 55.87, 54.96, 38.58, 30.99, 17.79, 15.66. MS-ESI (*m*/*z*): 453 [M + H]^+^.

*(E)-N-(2-(3-(3,4-Dichlorophenyl)acrylamido)ethyl)-2,6-dimethylimidazo[1,2-a]pyridine-3-carboxamide* (**11c**): The title compound was prepared from **5c** and **9** as a white solid (57%); m.p.: 213–215 °C. ^1^H-NMR (500 MHz, DMSO-*d*_6_) δ (ppm): 8.82 (s, 1H, pyridine-H), 8.37 (m, 1H, -CONH-), 7.88–7.83 (m, 2H, -CONH- and Ar-H), 6.65 (d, *J* = 8.5 Hz, 1H, Ar-H), 7.55 (dd, *J* = 8.0, 1.5 Hz, 1H, Ar-H), 7.46 (d, *J* = 9.0 Hz, 1H, pyridine-H), 7.42 (d, *J* = 16.0 Hz, 1H, =C-H), 7.22 (dd, *J* = 9.5, 2.0 Hz, 1H, Ar-H), 6.72 (d, *J* = 16.0 Hz, 1H, =C-H), 3.44 ( m, 4H, 2 × -CH2-), 2.55 (s, 3H, CH3), 2.27 (s, 3H, CH3). ^13^C-NMR (126 MHz, DMSO-*d*_6_) δ (ppm): 164.94, 161.16, 144.92, 143.82, 136.16, 135.84, 131.69, 131.61, 131.06, 129.44, 129.17, 127.28, 124.78, 124.44, 121.94, 115.67, 115.46, 38.86, 38.72, 17.79, 15.66. MS-ESI (*m*/*z*): 431 [M + H]^+^.

*(E)-N-(2-(3-(4-Methoxyphenyl)acrylamido)ethyl)-2,6-dimethylimidazo[1,2-a]pyridine-3-carboxamide* (**11d**): The title compound was prepared from **5d** and **9** as a white solid (69%); m.p.: 203–205 °C. ^1^H-NMR (500 MHz, CDCl3) δ (ppm): 9.12 (s, 1H, pyridine-H), 7.55 (d, *J* = 15.5 Hz, 1H, =C-H), 7.44 (d, *J* = 9.0 Hz, 1H, pyridine-H), 7.40 (d, *J* = 9.0 Hz, 1H, pyridine-H), 7.14 (dd, *J* = 9.0, 1.5 Hz, 1H, Ar-H), 6.85 (d, *J* = 9.0 Hz, 1H, Ar-H), 6.80 (s, 1H, -CONH-), 6.60 (s, 1H, -CONH-), 6.29 (d, *J* = 15.5 Hz, 1H, =C-H), 3.80 (s, 3H, OCH3), 3.69–3.67 (m, 4H, 2 × -CH2-), 2.71 (s, 3H, CH3), 2.31 (s, 3H, CH3). ^13^C-NMR (126 MHz, CDCl3) δ (ppm): 167.73, 162.64, 161.11, 145.81, 144.99, 145.37, 130.11, 129.55, 127.38, 125.96, 123.10, 117.73, 115.71, 115.21, 114.35, 55.48, 41.06, 40.12, 18.49, 16.59. MS-ESI (*m*/*z*): 393 [M + H]^+^.

*(E)-2,6-Dimethyl-N-(2-(3-(4-(trifluoromethyl)phenyl)acrylamido)ethyl)imidazo[1,2-a]pyridine-3-carboxamide* (**11e**): The title compound was prepared from **5e** and **9** as a white solid (72%); m.p.: 252–254 °C. ^1^H-NMR (500 MHz, DMSO-*d*_6_) δ (ppm): 8.82 (s, 1H, pyridine-H), 8.82–8.40 (m, 1H, Ar-H), 7.87–7.85 (m, 1H, Ar-H), 7.77 (dd, *J* = 9.0, 11.5 Hz, 1H, Ar-H), 7.52 (d, *J* = 16.0 Hz, 1H, =C-H), 7.46 (d, *J* = 9.0 Hz, 1H, pyridine-H), 7.22 (dd, *J* = 9.0, 1.5 Hz, 1H, Ar-H), 6.77 (d, *J* = 16.0 Hz, 1H, =C-H), 3.46–3.43 (m, 4H, 2 × -CH_2_-), 2.55 (s, 3H, CH_3_), 2.26 (s, 3H, CH_3_). ^13^C-NMR (126 MHz, DMSO-*d*_6_) δ (ppm): 164.91, 161.16, 144.92, 143.83, 138.98, 137.08, 129.33, 129.17, 128.17, 125.84, 125.81, 125.21, 124.95, 124.76, 121.93, 115.68, 115.49, 38.87, 38.62, 17.78, 15.65. HRMS-ESI (*m/z*): calcd. for C_22_H_22_O_2_N_4_F_3_ [M + H]^+^: 431.1695; found 431.1675.

*(E)-N-(2-(3-(2-Fluorophenyl)acrylamido)ethyl)-2,6-dimethylimidazo[1,2-a]pyridine-3-carboxamide* (**11f**): The title compound was prepared from **5f** and **9** as a white solid (70%); m.p.: 197–199 °C. ^1^H-NMR (500 MHz, DMSO-*d*_6_) δ (ppm): 8.82 (s, 1H, pyridine-H), 8.42 (t, *J* = 5.0 Hz, 1H, -CONH-), 7.86 (t, *J* = 5.0 Hz, 1H, -CONH-), 7.67–7.64 (m, 1H, Ar-H), 7.52–7.40 (m, 3H, =C-H and Ar-H), 7.30–7.21 (m, 3H, pyridine-H and Ar-H), 6.74 (d, *J* = 16.0 Hz, 1H, =C-H), 3.45–3.42 (m, 4H, 2 × -CH_2_-), 2.54 (s, 3H, CH_3_), 2.28 (s, 3H, CH_3_). ^13^C-NMR (126 MHz, DMSO-*d*_6_) δ (ppm): 165.13, 161.48, 161.15, 159.49, 144.90, 143.82, 131.37, 131.30, 131.16, 129.22, 129.20, 129.18, 125.05, 124.99, 124.77, 121.94, 116.21, 116.04, 115.71, 115.49, 38.87, 38.60, 17.77, 15.62. MS-ESI (*m*/*z*): 381 [M + H]^+^.

*(E)-N-(2-(3-(4-Fluorophenyl)acrylamido)ethyl)-2,6-dimethylimidazo[1,2-a]pyridine-3-carboxamide* (**11g**): The title compound was prepared from **5g** and **9** as a white solid (67%); m.p.: 208–211 °C. ^1^H-NMR (500 MHz, DMSO-*d*_6_) δ (ppm): 9.14 (s, 1H, pyridine-H), 7.55 (d, *J* = 15.5 Hz, 1H, =C-H), 7.46–7.42 (m, 3H, pyridine-H and Ar-H), 7.04 (dd, *J* = 9.0, 1.5 Hz, 1H, Ar-H), 7.05–7.01 (m, 2H, Ar-H), 6.67 (s, 1H, -CONH-), 6.60 (s, 1H, -CONH-), 6.34 (d, *J* = 15.5 Hz, 1H, =C-H), 3.72–3.67 (m, 4H, 2 × -CH_2_-), 2.72 (s, 3H, CH_3_), 2.32 (s, 3H, CH_3_). ^13^C-NMR (126 MHz, DMSO-*d*_6_) δ (ppm): 162.12, 159.72, 157.81, 157.73, 140.98, 140.17, 135.50, 125.94, 125.92, 125.11, 124.81, 124.74, 120.97, 118.11, 114.94, 114.92, 111.16, 110.99, 110.84, 110.09, 35.81, 35.41, 13.52, 11.74. MS-ESI (*m*/*z*): 381 [M + H]^+^.

*(E)-N-(2-(3-(3-Fluorophenyl)acrylamido)ethyl)-2,6-dimethylimidazo[1,2-a]pyridine-3-carboxamide* (**11h**): The title compound was prepared from **5h** and **9** as a white solid (65%); m.p.: 172–175 °C. ^1^H-NMR (500 MHz, DMSO-*d*_6_) δ (ppm): 8.83 (s, 1H, pyridine-H), 8.32 (d, *J* = 5.5 Hz, 1H, -CONH-), 7.83 (d, *J* = 5.5 Hz, 1H, -CONH-), 7.46–7.39 (m, 5H, Ar-H), 7.22–7.19 (m, 2H, Ar-H and pyridine-H), 6.34 (d, *J* = 15.5 Hz, 1H, =C-H), 3.47–3.36 (m, 4H, 2 × -CH_2_-), 2.55 (s, 3H, CH_3_), 2.27 (s, 3H, CH_3_). ^13^C-NMR (126 MHz, DMSO-*d*_6_) δ (ppm): 165.12, 163.45, 161.51, 161.18, 144.93, 143.84, 137, 55, 137.49, 130.96, 130.89, 129.17, 124, 78, 123.71, 121.95, 116.24, 116.07, 115.69, 115.49, 114.05, 113.88, 38.91, 38.62, 17.78, 15.65. MS-ESI (*m*/*z*): 381 [M + H]^+^.

*(E)-N-(2-(3-(4-Chlorophenyl)acrylamido)ethyl)-2,6-dimethylimidazo[1,2-a]pyridine-3-carboxamide* (**11i**): The title compound was prepared from **5i** and **9** as a white solid (59%); m.p.: 252–254 °C. ^1^H-NMR (500 MHz, DMSO-*d*_6_) δ (ppm): 8.82 (s, 1H, pyridine-H), 8.31 (s, 1H, -CONH-), 7.83 (s, 1H, -CONH-), 7.59 (d, *J* = 8.5 Hz, 2H, Ar-H), 7.48–7.40 (m, 4H, Ar-H and pyridine-H), 7.23 (dd, *J* = 9.0, 1.5 Hz, 1H, Ar-H), 6.64 (d, *J* = 16.0 Hz, 1H, =C-H), 3.45–3.41 (m, 4H, 2 × -CH_2_-), 2.54 (s, 3H, CH_3_), 2.28 (s, 3H, CH_3_). ^13^C-NMR (126 MHz, DMSO-*d*_6_) δ (ppm): 165.19, 161.15, 144.91, 143.82, 137.40, 133.90, 133.86, 129.25, 129.18, 128.99, 124.77, 122.95, 121.95, 115.69, 115.48, 38.93, 38.57, 17.79, 15.64. MS-ESI (*m*/*z*): 397 [M + H]^+^.

*(E)-2,6-Dimethyl-N-(2-(3-(4-nitrophenyl)acrylamido)ethyl)imidazo[1,2-a]pyridine-3-carboxamide* (**11J**): The title compound was prepared from **5J** and **9** as a white solid (52%); m.p.: 213–215 °C. ^1^H-NMR (500 MHz, DMSO-*d*_6_) δ (ppm): 8.82 (s, 1H, pyridine-H), 8.48 (s, 1H, -CONH-), 8.24 (d, *J* = 9.0 Hz, 2H, pyridine-H), 7.87–7.82 (m, 3H, -CONH- and Ar-H), 7.55 (d, *J* =15.5 Hz, 1H, =C-H), 7.45 (d, *J* = 9.0 Hz, 2H, pyridine-H) 7.21 (dd, *J* = 9.0, 1.5 Hz, 1H, Ar-H), 6.84 (d, *J* = 16.0 Hz, 1H, =C-H), 3.47–3.43 (m, 4H, 2 × -CH_2_-), 2.55 (s, 3H, CH_3_), 2.27 (s, 3H, CH_3_). ^13^C-NMR (126 MHz, DMSO-*d*_6_) δ (ppm): 164.70, 161.16, 147.49, 144.94, 143.83, 141.51, 136.43, 129.16, 128.59, 126.43, 124.76, 124.13, 121.94, 115.66, 115.48, 38.82, 38.68, 17.80, 15.66. MS-ESI (*m*/*z*): 408 [M + H]^+^.

*(E)-2,6-Dimethyl-N-(2-(3-(4-(trifluoromethoxy)phenyl)acrylamido)ethyl)imidazo[1,2-a]pyridine-3-carboxamide* (**11k**): The title compound was prepared from **5k** and **9** as a white solid (64%); m.p.: 214–217 °C. ^1^H-NMR (500 MHz, DMSO-*d*_6_) δ (ppm): 8.82 (s, 1H, pyridine-H), 8.81–8.42 (m, 1H, -CONH-), 7.85–7.82 (m, 1H, -CONH-), 7.70 (dd, *J* = 9.0, 11.5 Hz, 1H, Ar-H), 7.58 (d, *J* = 16.0 Hz, 1H, =C-H), 7.48 (d, *J* = 9.0 Hz, 1H, pyridine-H), 7.23 (dd, *J* = 9.0, 1.5 Hz, 1H, Ar-H), 6.97 (d, *J* = 16.0 Hz, 1H, =C-H), 3.50–3.45 (m, 4H, 2 × -CH_2_-), 2.58 (s, 3H, CH_3_), 2.24 (s, 3H, CH_3_). ^13^C-NMR (126 MHz, DMSO-*d*_6_) δ (ppm): 165.90, 162.15, 145.82, 143.83, 139.92, 137.18, 129.32, 129.17, 128.18, 126.85, 125.98, 125.31, 124.85, 124.74, 122.43, 114.90, 114.49, 38.85, 37.65, 18.75, 15.65. HRMS-ESI (*m*/*z*): calcd. for C_22_H_22_O_3_N_4_F_3_ [M + H]^+^: 447.1644; found 447. 1622.

*(E)-N-(2-Cinnamamidoethyl)-2,6-dimethylimidazo[1,2-a]pyridine-3-carboxamide* (**11l**): The title compound was obtained from **5l** and **9** as a white solid (58%); m.p.: 196–198 °C. ^1^H-NMR (500 MHz, CDCl_3_) δ (ppm): 9.22 (s, 1H, pyridine-H), 7.65 (d, *J* = 16.0 Hz, 1H, =C-H), 7.52–7.47 (m, 3H, Ar-H and pyridine-H), 7.38–7.36 (m, 3H, Ar-H and pyridine-H), 7.18 (dd, *J* = 9.0, 2.0 Hz, 1H, Ar-H), 6.89 (t, *J* = 6.0 Hz, 1H, -CONH-), 6.58 (t, *J* = 6.0 Hz, 1H, -CONH-), 6.48 (d, *J* = 16.0 Hz, 1H, =C-H), 3.59 (dd, *J* = 12.0, 6.5 Hz, 2H, -CH_2_-), 3.54 (dd, *J* = 12.0, 6.5 Hz, 2H, -CH_2_-), 2.83 (s, 3H, CH_3_), 2.37 (s, 3H, CH_3_). ^13^C-NMR (126 MHz, CDCl_3_) δ (ppm): 166.96, 162.28, 145.66, 145.03, 141.40, 134.81, 129.97, 129.88, 128.94, 127.93, 126.04, 123.00, 120.55, 115.76, 115.39, 36.26, 35.66, 30.53, 18.52, 16.79. MS-ESI (*m*/*z*): 363 [M + H]^+^.

*(E)-2,7-Dimethyl-N-(2-(3-p-tolylacrylamido)ethyl)imidazo[1,2-a]pyridine-3-carboxamide* (**12a**): The title compound was prepared from **5a** and **10** as a white solid (61%); m.p.: 244–247 °C. ^1^H-NMR (500 MHz, DMSO-*d*_6_) δ (ppm): 8.94 (d, *J* = 7.5 Hz, 1H, pyridine-H), 8.28 (d, *J* = 5.0 Hz, 1H, -CONH-), 7.79 (d, *J* = 5.0 Hz, 1H, -CONH-), 7.45 (d, *J* = 8.0 Hz, 2H, Ar-H), 7.40 (d, *J* = 15.5 Hz, 1H, =C-H), 7.34–7.33 (m, 1H, Ar-H and pyridine-H), 7.22 (d, *J* = 8.0 Hz, 2H, Ar-H), 6.58 (d, *J* = 15.5 Hz, 1H, =C-H), 3.45–3.41 (m, 4H, 2 × -CH_2_-), 2.55 (s, 3H, CH_3_), 2.36 (s, 3H, CH_3_), 2.32 (s, 3H, CH_3_). ^13^C-NMR (126 MHz, DMSO-*d*_6_) δ (ppm): 165.55, 161.15, 145.27, 145.16, 139.23, 138.71, 137.12, 132.12, 129.55, 127.52, 126.43, 121.08, 115.33, 115.15, 114.54, 38.52, 20.96, 20.73, 15.71. MS-ESI (*m*/*z*): 377 [M + H]^+^.

*(E)-2,7-Dimethyl-N-(2-(3-(3,4,5-trimethoxyphenyl)acrylamido)ethyl)imidazo[1,2-a]pyridine-3-carboxamide* (**12b**): The title compound was obtained from **5b** and **10** as a white solid (61%); m.p.: 225–227 °C. ^1^H-NMR (500 MHz, DMSO-*d*_6_) δ (ppm): 8.92 (d, *J* = 7.0 Hz, 1H, pyridine-H), 8.24 (t, *J* = 5.0 Hz, 1H, -CONH-), 7.85 (t, *J* = 5.0 Hz, 1H, -CONH-), 7.47 (d, *J* = 9.0 Hz, 1H, pyridine-H), 7.38 (d, *J* = 15.5 Hz, 1H, =C-H), 7.23 (dd, *J* = 7.0, 1.5 Hz, 1H, Ar-H), 6.90 (s, 2H, Ar-H), 6.60 (d, *J* = 15.5 Hz, 1H, =C-H), 3.80 (s, 6H, 2 × OCH_3_), 3.67 (s, 3H, OCH_3_), 3.44–3.41 (m, 4H, 2 × -CH_2_-), 2.55 (s, 3H, CH_3_), 2.28 (s, 3H, CH_3_). ^13^C-NMR (126 MHz, DMSO-*d*_6_) δ (ppm): 166.38, 162.14, 154.10, 145.12, 143.83, 138.94, 138.50, 131.40, 128.19, 124.68, 122.14, 121.45, 115.67, 115.40, 105.81, 61.15, 56.88, 53.97, 39.57, 31.87, 18.80, 15.64. MS-ESI (*m*/*z*): 453 [M + H]^+^.

*(E)-N-(2-(3-(3,4-Dichlorophenyl)acrylamido)ethyl)-2,7-dimethylimidazo[1,2-a]pyridine-3-carboxamide* (**12c**): The title compound was prepared from **5c** and **10** as a white solid (54%); m.p.: 201–204 °C. ^1^H-NMR (500 MHz, DMSO-*d*_6_) δ (ppm): 8.92 (d, *J* = 7.5 Hz, 1H, pyridine-H), 8.34 (d, *J* = 5.5 Hz, 1H, -CONH-), 7.84 (d, *J* = 2.0 Hz, 1H, pyridine-H), 7.78 (d, *J* = 5.0 Hz, 1H, -CONH-), 7.66 (d, *J* = 8.0, 1H, pyridine-H), 7.55 (dd, *J* = 8.5, 2.0 Hz, 1H, Ar-H), 7.42 (d, *J* = 16.0 Hz, 1H, =C-H), 7.32 (s, 1H, Ar-H), 6.82 (dd, *J* = 7.5, 2.0 Hz, 1H, Ar-H), 6.70 (d, *J* = 16.0 Hz, 1H, =C-H), 3.43 ( m, 4H, 2 × -CH_2_-), 2.55 (s, 3H, CH_3_), 2.35 (s, 3H, CH_3_). ^13^C-NMR (126 MHz, DMSO-*d*_6_) δ (ppm): 164.93, 161.16, 145.26, 145.15, 137.15, 136.19, 135.84, 131.69, 131.61, 131.07, 129.48, 127.28, 126.44, 124.42, 115.32, 115.16, 114.53, 38.87, 38.63, 20,72, 15.72. MS-ESI (*m*/*z*): 431 [M + H]^+^.

*(E)-N-(2-(3-(4-Methoxyphenyl)acrylamido)ethyl)-2,7-dimethylimidazo[1,2-a]pyridine-3-carboxamide* (**12d**): The title compound was prepared from **5d** and **10** as a white solid (67%); m.p.: 218–221 °C. ^1^H-NMR (500 MHz, DMSO-*d*_6_) δ (ppm): 8.94 (d, *J* = 7.0 Hz, 1H, pyridine-H), 8.21 (d, *J* = 5.0 Hz, 1H, -CONH-), 7.78 (d, *J* = 5.0 Hz, 1H, -CONH-), 7.79–7.77 (m, 2H, Ar-H and pyridine-H), 7.34–7.37 (m, 2H, Ar-H and pyridine-H), 6.98–6.95 (m, 2H, Ar-H), 6.86–6.84 (m, 2H, Ar-H), 6.48 (d, *J* = 16.0 Hz, 1H, =C-H), 3.78 (s, 3H, OCH_3_), 3.44–3.40 (m, 4H, 2 × -CH_2_-), 2.55 (s, 3H, CH_3_), 2.36 (s, 3H, CH_3_). ^13^C-NMR (126 MHz, CDCl_3_) δ (ppm): 165.72, 161.13, 160.34, 145.27, 145.15, 138.47, 137.13, 129.13, 127.43, 126.44, 119.62, 115.34, 115.16, 114.55, 114.40, 55.27, 38.49, 20.73, 15.70. MS-ESI (*m*/*z*): 393 [M + H]^+^.

*(E)-2,7-Dimethyl-N-(2-(3-(4-(trifluoromethyl)phenyl)acrylamido)ethyl)imidazo[1,2-a]pyridine-3-carboxamide* (**12e**): The title compound was prepared from **5e** and **10** as a white solid (72%); m.p.: 218–221 °C. ^1^H-NMR (500 MHz, DMSO-*d*_6_) δ (ppm): 8.83 (s, 1H, pyridine-H), 8.38 (t, *J* = 5.0, 1H, -CONH-), 7.83 (t, *J* = 5.0, 1H, -CONH-) 7.79–7.75 (m, 4H, Ar-H and pyridine-H), 7.51 (d, *J* = 16.0 Hz, 1H), 7.46 (d, *J* = 9.0 Hz, 1H, Ar-H), 7.22 (dd, *J* = 9.0, 1.5 Hz, 1H, Ar-H), 6.77 (d, *J* = 16.0 Hz, 1H, =C-H), 3.46–3.43 (m, 4H, 2 × -CH_2_-), 2.55 (s, 3H, CH_3_), 2.27 (s, 3H, CH_3_). ^13^C-NMR (126 MHz, DMSO-*d*_6_) δ (ppm): 165.71, 161.13, 160.34, 145.27, 145.15, 138.47, 137.13, 129.13, 127.43, 126.44, 119.62, 115.34, 115.16, 114.54, 114.40, 55.27, 38.49, 20.73, 15.70. MS-ESI (*m*/*z*): 431 [M + H]^+^.

*(E)-N-(2-(3-(2-Fluorophenyl)acrylamido)ethyl)-2,7-dimethylimidazo[1,2-a]pyridine-3-carboxamide* (**12f**): The title compound was prepared from **5f** and **10** as a white solid (61%); m.p.: 213–216 °C. ^1^H-NMR (500 MHz, DMSO-*d*_6_) δ (ppm): 8.92 (d, *J* = 7.0 Hz, 1H, pyridine-H), 8.44 (d, *J* = 5.0 Hz, 1H, -CONH-), 7.79 (d, *J* = 5.0 Hz, 1H, -CONH-), 7.67–7.63 (m, 1H, Ar-H), 7.51 (d, *J* = 16.0 Hz, 1H, =C-H), 7.43–7.40 (m, 1H, Ar-H), 7.32 (s, 1H), 7.29–7.23 (m, 2H, Ar-H), 6.81 (dd, *J* = 7.5, 1.5 Hz, 1H), 6.74 (d, *J* = 16.0 Hz, 1H, =C-H), 3.45–3.42 (m, 4H, 2 × -CH_2_-), 2.55 (s, 3H, CH_3_), 2.35 (s, 3H, CH_3_). ^13^C-NMR (126 MHz, CDCl_3_) δ (ppm): 165.59, 161.92, 161.60, 159.93, 145.72, 145.60, 137.56, 131.79, 131.61, 129.65, 129.63, 126.87, 125.47, 125.44, 125.42, 116.64, 116.47, 115.78, 115.57, 114.98, 39.34, 39.05, 21.16, 16.14. MS-ESI (*m*/*z*): 381 [M + H]^+^.

*(E)-N-(2-(3-(4-Fluorophenyl)acrylamido)ethyl)-2,7-dimethylimidazo[1,2-a]pyridine-3-carboxamide* (**12g**): The title compound was prepared from **5g** and **10** as a white solid (65%); m.p.: 246–248 °C. ^1^H-NMR (500 MHz, DMSO-*d*_6_) δ (ppm): 8.93 (d, *J* = 7.5 Hz, 1H, pyridine-H), 8.30 (t, *J* = 5.0 Hz, 1H, -CONH-), 7.62 (t, *J* = 3.5 Hz, 1H, -CONH-), 7.64–7.61 (m, 1H, Ar-H), 7.43 (d, *J* = 16.0 Hz, 1H, =C-H), 7.33 (s, 1H, Ar-H), 7.27–7.23 (m, 2H, Ar-H and pyridine-H), 6.83 (dd, *J* = 7.0, 1.5 Hz, 1H), 6.57 (d, *J* = 16.0 Hz, 1H, =C-H), 3.44–3.40 (m, 4H, 2 × -CH_2_-), 2.54 (s, 3H, CH_3_), 2.36 (s, 3H, CH_3_). ^13^C-NMR (126 MHz, CDCl_3_) δ (ppm): 165.77, 164.13, 162.17, 161.59, 145.71, 145.59, 138.02, 137.60, 131.98, 131.95, 130.19, 130.12, 126.88, 122.47, 116.47, 116.30, 115.78, 115.62, 114.98, 39.41, 38.99, 21.18, 16.15. MS-ESI (*m*/*z*): 381 [M + H]^+^.

*(E)-N-(2-(3-(3-Fluorophenyl)acrylamido)ethyl)-2,7-dimethylimidazo[1,2-a]pyridine-3-carboxamide* (**12h**): The title compound was prepared from **5h** and **10** as a white solid (50%); m.p.: 172–175 °C. ^1^H-NMR (500 MHz, DMSO-*d*_6_) δ (ppm): 8.93 (d, *J* = 7.5 Hz, 1H, pyridine-H), 8.33 (t, *J* = 5.0 Hz, 1H, -CONH-), 7.78 (t, *J* = 5.0 Hz, 1H, -CONH-), 7.48-7.40 (m, 4H, Ar-H and pyridine-H), 7.33 (s, 1H, Ar-H), 7.23–7.18 (m, 1H, Ar-H), 6.83 (dd, *J* = 7.0, 1.5 Hz, 1H, Ar-H), 6.58 (d, *J* = 16.0 Hz, 1H, =C-H), 3.44–3.41 (m, 4H, 2 × -CH_2_-), 2.55 (s, 3H, CH_3_), 2.36 (s, 3H, CH_3_). ^13^C-NMR (126 MHz, DMSO-*d*_6_) δ (ppm): 165.10, 163.44, 161.51, 161.16, 145.28, 145.17, 137.55, 137.48, 137.14, 130.96, 130.89, 126.44, 123.70, 116.24, 116.07, 115.33, 115.16, 114.55, 114.06, 113.89, 38.91, 38.59, 20.73, 15.71. MS-ESI (*m*/*z*): 381 [M + H]^+^.

*(E)-N-(2-(3-(4-Chlorophenyl)acrylamido)ethyl)-2,7-dimethylimidazo[1,2-a]pyridine-3-carboxamide* (**12i**): The title compound was prepared from **5i** and **10** as a white solid (61%); m.p.: 172–175 °C. ^1^H-NMR (500 MHz, DMSO-*d*_6_) δ (ppm): 8.83 (s, 1H, pyridine-H), 8.32 (d, *J* = 5.5 Hz, 1H, -CONH-), 7.83 (d, *J* = 5.0 Hz, 1H, -CONH-), 7.46–7.39 (m, 5H, pyridine-H, Ar-H and =C-H), 7.21 (d, *J* = 9.0 Hz, 1H, Ar-H), 6.69 (d, *J* = 15.5 Hz, 1H, =C-H, Ar-H), 3.47–3.43 (m, 4H, 2 × -CH_2_-), 2.55 (s, 3H, CH_3_), 2.27 (s, 3H, CH_3_). ^13^C-NMR (126 MHz, DMSO-*d*_6_) δ (ppm): 165.56, 161.60, 149.25, 145.72, 145.61, 137.59, 134.72, 129.89, 129.79, 126.88, 123.80, 121.91, 121.51, 119,47, 115.77, 115.61, 114.99, 39.36, 39.01, 31.17, 13.15. MS-ESI (*m*/*z*): 397 [M + H]^+^.

*(E)-2,7-Dimethyl-N-(2-(3-(4-nitrophenyl)acrylamido)ethyl)imidazo[1,2-a]pyridine-3-carboxamide* (**11j**): The title compound was prepared from **5j** and **10** as a white solid (71%); m.p.: 213–215 °C. ^1^H-NMR (500 MHz, DMSO-*d*_6_) δ (ppm): 8.93 (d, *J* = 7.0 Hz, 1H, pyridine-H), 8.45 (s, 1H, -CONH-), 8.25 (d, *J* = 8.5 Hz, 1H, Ar-H), 7.83 (d, *J* = 8.5 Hz, 1H, Ar-H), 7.78 (d, *J* = 5.5 Hz, 1H, -CONH-), 7.55 (d, *J* = 16.0 Hz, 1H, =C-H, Ar-H), 7.33 (s, 1H, Ar-H), 6.85–6.80 (m, 2H, Ar-H), 3.44 (s, 4H, 2 × -CH_2_-), 2.54 (s, 3H, CH_3_), 2.36 (s, 3H, CH_3_). ^13^C-NMR (126 MHz, DMSO-*d*_6_) δ (ppm): 164.69, 161.17, 147.52, 145.28, 145.18, 141.51, 137.15, 136.46, 128.62, 126.43, 124.16, 115.31, 115.17, 114.55, 38.83, 38.66, 20.73, 15.72. MS-ESI (*m*/*z*): 408 [M + H]^+^.

*(E)-2,6-Dimethyl-N-(2-(3-(4-(trifluoromethoxy)phenyl)acrylamido)ethyl)imidazo[1,2-a]pyridine-3-carboxamide* (**12k**): The title compound was prepared from **5k** and **10** as a white solid (61%); m.p.: 214–216 °C. ^1^H-NMR (500 MHz, DMSO-*d*_6_) δ (ppm): 8.93 (d, *J* = 7.0 Hz, 1H, pyridine-H), 8.33 (d, *J* = 5.0 Hz, 1H, -CONH-), 7.78 (d, *J* = 5.0 Hz, 1H, -CONH-), 7.59 (dd, *J* = 7.0, 2.0 Hz, 2H, Ar-H), 7.45 (dd, *J* = 7.0, 2.0 Hz, 2H, Ar-H), 7.43 (d, *J* = 16.0 Hz, 1H, =C-H), 7.33 (s, 1H, pyridine-H), 6.83 (dd, *J* = 7.5, 2.0 Hz, 1H, pyridine-H), 6.63 (d, *J* = 16.0 Hz, 1H, =C-H), 3.44–3.40 (m, 4H, 2 × -CH_2_-), 2.54 (s, 3H, CH_3_), 2.36 (s, 3H, CH_3_). ^13^C-NMR (126 MHz, DMSO-*d*_6_) δ (ppm): 165.17, 161.15, 145.27, 145.16, 137.40, 137.14, 133.89, 133.85, 129.26, 129.00, 126.43, 122.94, 115.32, 115.16, 114.55, 54.96, 38.56, 20.73, 15.71. HRMS-ESI (*m*/*z*): calcd. for C_22_H_22_O_3_N_4_F_3_ [M + H]^+^: 447.1644; found 447. 1631.

*(E)-N-(2-Cinnamamidoethyl)-2,7-dimethylimidazo[1,2-a]pyridine-3-carboxamide* (**12l**): The title compound was obtained from **5l** and **10** as a white solid (59%); m.p.: 197–200 °C. ^1^H-NMR (500 MHz, DMSO-*d*_6_) δ (ppm): 8.93 (d, *J* = 7.0 Hz, 1H, pyridine-H), 8.30 (d, *J* = 5.0 Hz, 1H, -CONH-), 7.56 (d, *J* = 5.0 Hz, 1H, -CONH-), 7.56–7.55 (m, 3H, Ar-H and pyridine-H), 7.42–7.34 (m, 3H, Ar-H), 6.85 (dd, *J* = 7.0, 2.0 Hz, 1H, pyridine-H), 6.63 (d, *J* = 14.5 Hz, 1H, =C-H), 3.45–3.41 (m, 4H, 2 × -CH_2_-), 2.55 (s, 3H, CH_3_), 2.37 (s, 3H, CH_3_). ^13^C-NMR (126 MHz, CDCl_3_) δ (ppm): 165.36, 161.13, 145.26, 145.14, 138.73, 137.13, 134.87, 129.49, 128.97, 127.54, 126.43,122.12, 115.33, 115.16, 114.54, 44.47, 38.53, 20.73, 15.70. MS-ESI (*m*/*z*): 363 [M + H]^+^.

*(E)-2,6-Dimethyl-N-(3-(3-p-tolylacrylamido)propyl)imidazo[1,2-a]pyridine-3-carboxamide* (**13a**): The title compound was obtained from **6a** and **9** as a white solid (52%); m.p.: 196–198 °C. ^1^H-NMR (500 MHz, CDCl_3_) δ (ppm): 9.15 (s, 1H, pyridine-H), 7.58 (d, *J* = 15.5 Hz, 1H, =C-H), 7.43 (d, *J* = 9.0 Hz, 2H, Ar-H), 7.34 (d, *J* = 8.0 Hz, 1H, pyridine-H), 7.13–7.11 (m, 3H, Ar-H and pyridine-H), 6.99 (t, *J* = 6.0 Hz, 1H, -CONH-), 6.81 (t, *J* = 6.0 Hz, 1H, -CONH-), 6.43 (d, *J* = 15.5 Hz, 1H, =C-H),3.55 (dd, *J* = 12.0, 6.0 Hz, 2H, -CH_2_-), 3.49 (dd, *J* = 12.0, 6.0 Hz, 2H, -CH_2_-), 2.79 (s, 3H, CH_3_), 2.32 (s, 6H, 2 × CH_3_), 1.81–1.78 (m, 2H, -CH_2_-). ^13^C-NMR (126 MHz, CDCl_3_) δ (ppm): 165.30, 160.92, 144.67, 143.79, 139.17, 138.58, 132.15, 129.54, 127.49, 124.70, 121.96, 121.12, 115.74, 115.49, 36.48, 29.56, 20.97, 17.82, 15.59. MS-ESI (*m*/*z*): 391 [M + H]^+^.

*(E)-2,6-Dimethyl-N-(3-(3-(3,4,5-trimethoxyphenyl)acrylamido)propyl)imidazo[1,2-a]pyridine-3-carboxamide* (**13b**): The title compound was obtained from **6b** and **9** as a white solid (48%); m.p.: 196–199 °C. ^1^H-NMR (500 MHz, CDCl_3_) δ (ppm): 9.12 (s, 1H, pyridine-H), 7.50 (d, *J* =15.5 Hz, 1H, =C-H), 7.40 (d, *J* =9.0 Hz, 1H, pyridine-H), 7.11 (dd, *J* = 9.0, 1.5 Hz, 1H, Ar-H), 6.97 (t, *J* = 6.0 Hz, 1H, -CONH-), 6.87 (t, *J* = 6.0 Hz, 1H, -CONH-), 6.66 (s, 2H, Ar-H), 6.39 (d, *J* = 15.5 Hz, 1H, =C-H), 3.82 (s, 3H, OCH_3_), 3.80 (s, 6H, 2 × OCH_3_), 3.54–3.45 (m, 4H, 2 × -CH_2_-), 2.76 (s, 3H, CH_3_), 2.29 (s, 3H, CH_3_), 1.79–1.74 (m, 2H, -CH_2_-). ^13^C-NMR (126 MHz, DMSO-*d*_6_) δ (ppm): 165.27, 160.94, 153.09, 144.70, 143.82, 138.81, 138.57, 130.56, 129.15, 124.71, 121.98, 121.52, 115.75, 115.50, 104.88, 60.10, 55.86, 36.48, 29.52, 17.82, 15.69. MS-ESI (*m*/*z*): 467 [M + H]^+^.

*(E)-N-(3-(3-(3,4-Dichlorophenyl)acrylamido)propyl)-2,6-dimethylimidazo[1,2-a]pyridine-3-carboxamide* (**13c**): The title compound was prepared from **6c** and **9** as a white solid (57%); m.p.: 213–215 °C. ^1^H-NMR (500 MHz, CDCl_3_) δ (ppm): ^1^H-NMR (500 MHz, CDCl_3_) δ (ppm): 9.17 (s, 1H, pyridine-H), 7.55–7.54 (m, 1H, Ar-H), 7.51–7.45 (m, 2H, Ar-H and pyridine-H), 7.41 (d, *J* = 8.5 Hz, 1H, Ar-H), 7.30–7.27 (m, 1H, Ar-H), 7.17 (d, *J* = 9.0 Hz, 1H, Ar-H), 6.83 (d, *J* = 5.0 Hz, 1H, -CONH-), 6.76 (d, *J* = 5.0 Hz, 1H, -CONH-), 6.44 (d, *J* = 15.5 Hz, 1H, =C-H), 3.58–3.48 (m, 4H, 2 × -CH_2_-), 2.78 (s, 3H, CH_3_), 2.35 (s, 3H, CH_3_), 1.85–1.80 (m, 2H, -CH_2_-). ^13^C-NMR (126 MHz, DMSO-*d*_6_) δ (ppm): 164.68, 160.95, 144.69, 143.80, 136.07, 135.88, 131.68, 131.06, 129.44, 129.14, 127.28, 124.70, 124.47, 121.96, 115.74, 115.50, 36.59, 36.49, 29.46, 17.82, 15.69. MS-ESI (*m*/*z*): 445 [M + H]^+^.

*(E)-N-(3-(3-(4-Methoxyphenyl)acrylamido)propyl)-2,6-dimethylimidazo[1,2-a]pyridine-3-carboxamide* (**13d**): The title compound was prepared from **6d** and **9** as a white solid (45%); m.p.: 178–181 °C. ^1^H-NMR (500 MHz, CDCl_3_) δ (ppm): 9.10 (s, 1H, pyridine-H), 7.53 (d, *J* = 15.5 Hz, 1H, =C-H), 7.39 (d, *J* = 9.0 Hz, 1H, Ar-H), 7.35 (d, *J* = 9.0 Hz, 1H, Ar-H), 7.10–7.05 (m, 2H, Ar-H and pyridine-H), 6.98 (t, *J* = 6.0 Hz, 1H, -CONH-), 6.79 (d, *J* = 8.5 Hz, 1H, -CONH-), 6.33 (d, *J* = 15.5 Hz, 1H, =C-H), 3.75 (s, 3H, OCH_3_), 3.53–3.44 (m, 4H, 2 × -CH_2_-), 2.76 (s, 3H, CH_3_), 2.83 (s, 3H, CH_3_), 1.79-1.74 (m, 2H, -CH_2_-). ^13^C-NMR (126 MHz, CDCl_3_) δ (ppm): 165.52, 160.94, 160.31, 144.71, 143.82, 138.38, 129.15, 129.11, 127.48, 124.73, 121.98, 119.67, 115.76, 115.50, 114.39, 55.26, 36.49, 36.46, 29.61, 17.82, 15.70. MS-ESI (*m*/*z*): 407 [M + H]^+^.

*(E)-2,6-Dimethyl-N-(3-(3-(4-(trifluoromethyl)phenyl)acrylamido)propyl)imidazo[1,2-a]pyridine-3-carboxamide* (**13e**): The title compound was prepared from **6e** and **9** as a white solid (32%); m.p.: 162–165 °C. ^1^H-NMR (500 MHz, CDCl_3_) δ (ppm): 9.19 (s, 1H, pyridine-H), 7.66–7.57 (m, 5H, pyridine-H and Ar-H), 7.46 (d, *J* = 9.5 Hz, 1H), 7.17 (dd, *J* = 9.0, 1.5 Hz, 1H, Ar-H), 6.76 (t, *J* = 6.0 Hz, 1H, -CONH-), 6.67 (d, *J* = 6.0 Hz, 1H, -CONH-), 6.54 (d, *J* = 15.5 Hz, 1H, =C-H), 3.60–3.50 (m, 4H, 2 × -CH_2_-), 2.80 (s, 3H, CH_3_), 2.35 (s, 3H, CH_3_), 1.86–1.81 (m, 2H, -CH_2_-). ^13^C-NMR (126 MHz, DMSO-*d*_6_) δ (ppm): 164.67, 161.50, 144.69, 143.80, 139.03, 136.98, 129.16, 128.17, 125.84, 125.81, 125.23, 124.97, 124.70, 121.98, 115.75, 115.51, 115.49, 36.60, 36.51, 29.46, 17.81, 15.68. MS-ESI (*m*/*z*): 445 [M + H]^+^.

*(E)-N-(3-(3-(2-Fluorophenyl)acrylamido)propyl)-2,6-dimethylimidazo[1,2-a]pyridine-3-carboxamide* (**13f**): The title compound was prepared from **6f** and **9** as a white solid (41%); m.p.: 197–199 °C. ^1^H-NMR (500 MHz, DMSO-*d*_6_) δ (ppm): 8.99 (s, 1H, pyridine-H), 8.42 (t, *J* = 5.0 Hz, 1H, -CONH-), 7.86 (t, *J* = 5.0 Hz, 1H, -CONH-), 7.67–7.64 (m, 1H, Ar-H), 7.53–7.41 (m, 3H, Ar-H and pyridine-H), 7.30–7.22 (m, 3H, =C-H and Ar-H), 6.74 (d, *J* = 16.0 Hz, 1H, =C-H), 3.45–3.42 (m, 4H, 2 × -CH_2_-), 2.55 (s, 3H, CH_3_), 2.29 (s, 3H, CH_3_), 1.85–1.82 (m, 2H, -CH_2_-). ^13^C-NMR (126 MHz, DMSO-*d*_6_) δ (ppm): 165.13, 161.48, 161.15, 159.49, 144.90, 143.82, 131.37, 131.30, 131.16, 129.22, 129.20, 129.18, 125.05, 124.99, 124.77, 121.94, 116.21, 116.04, 115.71, 115.49, 38.87, 38.60, 29.44, 17.77, 15.62. MS-ESI (*m*/*z*): 395 [M + H]^+^.

*(E)-N-(3-(3-(4-Fluorophenyl)acrylamido)propyl)-2,6-dimethylimidazo[1,2-a]pyridine-3-carboxamide* (**13g**): The title compound was prepared from **6g** and **9** as a white solid (67%); m.p.: 151–153 °C. ^1^H-NMR (500 MHz, DMSO-*d*_6_) δ (ppm): 8.84 (s, 1H, pyridine-H), 8.22 (t, *J* = 5.5 Hz, 1H, -CONH-), 7.84 (t, *J* = 5.5 Hz, 1H, -CONH-), 7.64–7.61 (m, 2H, pyridine-H and Ar-H), 7.47 (d, *J* = 9.5 Hz, 1H, Ar-H), 7.44 (d, *J* = 15.5 Hz, 1H, =C-H), 7.27–7.21 (m, 3H, pyridine-H and Ar-H), 6.59 (d, *J* = 15.5 Hz, 1H), 3.39–3.27 (m, 4H, 2 × -CH_2_-), 2.58 (s, 3H, CH_3_), 2.30 (s, 3H, CH_3_), 1.79–1.73 (m, 2H, -CH_2_-). ^13^C-NMR (126 MHz, DMSO-*d*_6_) δ (ppm): 165.54, 164.10, 162.14, 161.38, 145.14, 144.25, 137.90, 132.01, 130.15, 130.08, 129.58, 125.15, 122.51, 116.44, 116.27, 116.19, 115.94, 36.96, 36.94, 29.97, 18.25, 16.13. MS-ESI (*m*/*z*): 395 [M + H]^+^.

*(E)-2,7-Dimethyl-N-(3-(3-p-tolylacrylamido)propyl)imidazo[1,2-a]pyridine-3-carboxamide* (**14a**): The title compound was obtained from **6a** and **10** as a white solid (59%); m.p.: 197–200 °C. ^1^H-NMR (500 MHz, CDCl_3_) δ (ppm): 9.20 (d, *J* = 7.0 Hz, 1H, pyridine-H), 7.56 (d, *J* = 15.5 Hz, 1H, =C-H), 7.32 (d, *J* = 7.5 Hz, 1H, Ar-H), 7.10 (d, *J* = 8.0 Hz, 2H, Ar-H), 6.98 (s, 2H, Ar-H), 6.68 (s, 1H, -CONH-), 6.42 (d, *J* = 15.5 Hz, 1H, =C-H), 3.54-3.46 (m, 4H, 2 × -CH_2_-), 2.77 (s, 3H, CH_3_), 2.38 (s, 3H, CH_3_), 2.31 (s, 3H, CH_3_), 1.77 (s, 2H, -CH_2_-). ^13^C-NMR (126 MHz, DMSO-*d*_6_) δ (ppm): 165.34, 160.96, 145.26, 144.93, 139.18, 138.61, 137.07, 132.16, 129.54, 127.50, 126.38, 121.12, 115.44, 115.16, 114.56, 36.48, 29.59, 20.96, 20.73, 15.75. MS-ESI (*m*/*z*): 391 [M + H]^+^.

*(E)-2,7-Dimethyl-N-(3-(3-(3,4,5-trimethoxyphenyl)acrylamido)propyl)imidazo[1,2-a]pyridine-3-carboxamide* (**14b**): The title compound was obtained from **6b** and **10** as a white solid (41%); m.p.: 192–195 °C. ^1^H-NMR (500 MHz, CDCl_3_) δ (ppm): 9.19 (d, *J* = 7.0 Hz, 1H, pyridine-H), 7.50 (d, *J* = 15.5 Hz, 1H, =C-H), 7.26 (d, *J* = 3.5 Hz, 1H), 6.93–6.91 (m, 2H, Ar-H), 6.69–6.67 (m, 3H, Ar-H), 6.39 (d, *J* = 15.5 Hz, 1H, =C-H), 3.82 (s, 3H, OCH_3_), 3.80 (s, 6H, 2 × OCH_3_), 3.54–3.45 (m, 4H, 2 × -CH_2_-), 2.76 (s, 3H, CH_3_), 2.35 (s, 3H, CH_3_), 1.77–1.73 (m, 2H, -CH_2_-). ^13^C-NMR (126 MHz, DMSO-*d*_6_) δ (ppm): 165.27, 160.95, 153.08, 145.25, 144.93, 138.80, 138.57, 137.08, 130.56, 126.37, 121.52, 115.42, 115.17, 114.56, 104, 88, 60.10, 55.86, 36.47, 36.44, 29.54, 20.73, 15.75. MS-ESI (*m*/*z*): 467 [M + H]^+^.

*(E)-N-(3-(3-(3,4-Dichlorophenyl)acrylamido)propyl)-2,7-dimethylimidazo[1,2-a]pyridine-3-carboxamid*e (**14c**): The title compound was prepared from **6c** and **10** as a white solid (54%); m.p.: 178–181 °C. ^1^H-NMR (500 MHz, CDCl_3_) δ (ppm): 9.27 (d, *J* = 7.0 Hz, 1H, pyridine-H), 7.57 (d, *J* = 1.5 Hz, 1H, pyridine-H), 7.52 (d, *J* = 15.5 Hz, 1H, =C-H), 7.44 (d, *J* = 8.5 Hz, 1H, Ar-H), 7.33–7.28 (m, 2H, Ar-H and -CONH-), 6.78–6.76 (m, 2H, Ar-H and -CONH-), 6.47 (d, *J* = 15.5 Hz, 1H, =C-H), 3.61–3.50 (m, 4H, 2 × -CH_2_-), 2.80 (s, 3H, CH_3_), 2.43 (s, 3H, CH_3_), 1.87–1.82 (m, 2H, -CH_2_-). ^13^C-NMR (126 MHz, DMSO-*d*_6_) δ (ppm): 165.13, 161.40, 145.68, 145.36, 137.51, 136.51, 136.32, 132.11, 132.00, 131.49, 129.88, 127.73, 126.80, 124.89, 115.86, 115.60, 114.99, 37.03, 36.92, 29.92, 21.17, 16.18. MS-ESI (*m*/*z*): 445 [M + H]^+^.

*(E)-N-(3-(3-(4-Methoxyphenyl)acrylamido)propyl)-2,7-dimethylimidazo[1,2-a]pyridine-3-carboxamide* (**14d**): The title compound was prepared from **6d** and **10** as a white solid (67%); m.p.: 217–220 °C. ^1^H-NMR (500 MHz, CDCl_3_) δ (ppm): 9.18 (d, *J* = 7.0 Hz, 1H, pyridine-H),7.52 (d, *J* = 15.5 Hz, 1H, =C-H), 7.37–7.33 (m, 2H, Ar-H and pyridine-H), 7.25 (d, *J* = 7.5 Hz, pyridine-H), 7.03–6.98 (m, 2H, Ar-H and -CONH-), 6.81–6.78 (m, 2H, Ar-H and -CONH-), 6.67 (dd, *J* = 1.5, 7.5 Hz, 1H, Ar-H), 6.48 (d, *J* = 16.0 Hz, 1H, =C-H), 3.76 (s, 3H), 3.52–3.44 (m, 4H, 2 × -CH_2_-), 2.76 (s, 3H, CH_3_), 2.34 (s, 3H, CH_3_), 1.77–1.75 (m, 2H, -CH_2_-). ^13^C-NMR (126 MHz, DMSO-*d*_6_) δ (ppm): 165.50, 160.94, 160.31, 145.25, 144.92, 138.36, 137.07, 129.10, 127.47, 119.66, 115.43, 115.17, 114.56, 114.38, 114.40, 55.20, 36.46, 36.45, 29.62, 20.73, 15.74. MS-ESI (*m*/*z*): 407 [M + H]^+^.

*(E)-2,7-Dimethyl-N-(3-(3-(4-(trifluoromethyl)phenyl)acrylamido)propyl)imidazo[1,2-a]pyridine-3-carboxamide* (**14e**): The title compound was prepared from **6e** and **10** as a white solid (22%); m.p.: 222–225 °C. ^1^H-NMR (500 MHz, DMSO-*d*_6_) δ (ppm): 9.26 (d, *J* = 7.0 Hz, 1H, pyridine-H), 8.38 (s, 1H, -CONH-), 7.65–7.57 (m, 4H, c and -CONH-), 7.33 (s, 1H, pyridine-H), 6.77–6.65 (m, 3H, Ar-H), 6.52 (d, *J* = 15.5 Hz, 1H, =C-H), 3.59–3.51 (m, 4H, 2 × -CH_2_-), 2.80 (s, 3H, CH_3_), 2.42 (s, 3H, CH_3_), 1.83–1.80 (m, 2H, -CH_2_-). ^13^C-NMR (126 MHz, DMSO-*d*_6_) δ (ppm): 164.67, 161.50, 144.69, 143.80, 139.03, 136.98, 129.16, 128.17, 125.84, 125.81, 125.23, 124.97, 124.70, 121.98, 115.75, 115.51, 115.49, 36.60, 36.51, 29.46, 17.81, 15.68. MS-ESI (*m*/*z*): 445 [M + H]^+^.

*(E)-N-(3-(3-(2-Fluorophenyl)acrylamido)propyl)-2,7-dimethylimidazo[1,2-a]pyridine-3-carboxamide* (**14f**): The title compound was prepared from **6f** and **9** as a white solid (34%); m.p.: 197–199 °C. ^1^H-NMR (500 MHz, DMSO-*d*_6_) δ (ppm): 8.93 (d, *J* = 7.0 Hz, 1H, pyridine-H), 8.45 (d, *J* = 5.0 Hz, 1H, -CONH-), 7.78 (d, *J* = 5.0 Hz, 1H, -CONH-), 7.67–7.62 (m, 1H, Ar-H), 7.51 (d, *J* = 16.0 Hz, 1H, =C-H), 7.43–7.41 (m, 1H, Ar-H), 7.33 (s, 1H, pyridine-H), 7.29–7.23 (m, 2H, Ar-H), 6.81 (dd, *J* = 7.5, 1.5 Hz, 1H, Ar-H), 6.74 (d, *J* = 16.0 Hz, 1H, =C-H), 3.45–3.42 (m, 4H, 2 × -CH_2_-), 2.54 (s, 3H, CH_3_), 2.28 (s, 3H, CH_3_), 1.85–1.82 (m, 2H, -CH_2_-). ^13^C-NMR (126 MHz, DMSO-*d*_6_) δ (ppm): 165.19, 161.92, 161.60, 159.93, 145.72, 145.60, 137.55, 131.78, 131.61, 129.65, 129.63, 126.87, 125.47, 125.44, 125.42, 116.64, 116.47, 115.78, 115.57, 114.98, 38.88, 38.61, 29.44, 17.76, 15.62. MS-ESI (*m*/*z*): 395 [M + H]^+^.

*(E)-N-(3-(3-(4-Fluorophenyl)acrylamido)propyl)-2,7-dimethylimidazo[1,2-a]pyridine-3-carboxamide* (**14g**): The title compound was prepared from **6g** and **10** as a white solid (55%); m.p.: 203–206 °C. ^1^H-NMR (500 MHz, DMSO-*d*_6_) δ (ppm): 8.92 (d, *J* = 7.5 Hz, 1H, pyridine-H), 8.22 (d, *J* = 5.5 Hz, 1H, -CONH-), 7.77 (d, *J* = 5.5 Hz, 1H, -CONH-), 7.64–7.60 (m, 1H, Ar-H), 7.42 (d, *J* = 16.0 Hz, 1H, =C-H), 7.41 (s, 1H, pyridine-H), 7.26–7.22 (m, 2H, Ar-H), 6.84 (dd, *J* = 7.0, 1.5 Hz, 1H, Ar-H), 6.58 (d, *J* = 16.0 Hz, 1H, =C-H), 3.37–3.26 (m, 4H, 2 × -CH_2_-), 2.57 (s, 3H, CH_3_), 2.35 (s, 3H, CH_3_), 1.78–1.72 (m, 2H, -CH_2_-). ^13^C-NMR (126 MHz, CDCl_3_) δ (ppm): 165.10, 163.66, 161.70, 160.95, 145.25, 144.92, 137.45, 137.07, 131.57, 131.55, 129.72, 129.65, 122.08, 116.00, 115.83, 115.43, 115.16, 114.56, 36.51, 36.48, 29.55, 20.73, 15.74. MS-ESI (*m*/*z*): 395 [M + H]^+^.

## 4. Conclusions

In summary, a series of novel imidazo[1,2-*a*]pyridine amide-cinnamamide hybrids, **11**–**14**, linked via an alkyl carbon chain were designed, synthesized and evaluated for their *in vitro* anti-MTB activity. All of the target hybrids are less active than the two reference compounds against MTB H37Rv ATCC 27294, but the promising activity (MICs: 4 μg/mL) of two compounds, **11e** and **11k**, suggests that they may be selectively targeted to MTB growths and could be a good starting point for further studies, as well as to find new lead compounds with better anti-MTB activity. By the way, the further expansion of the imidazo[1,2-*a*]pyridine amide-cinnamamide hybrids is underway to find a potent anti-TB agent in our lab.

## Supplementary Materials

Supplementary materials can be accessed at: http://www.mdpi.com/1420-3049/21/1/49/s1.
