# Supplementary material for: Design, Synthesis and Antimycobacterial Activity of Novel Imidazo[1,2-a]pyridine Amide-Cinnamamide Hybrids
Source: Molecules. 2015 Dec 30;21(1):49. doi: 10.3390/molecules21010049 (PMC6273240; doi:10.3390/molecules21010049)
Supplement: Supplementary file 1 [file molecules-21-00049-s001.pdf]

Supplementary Materials: Design, Synthesis and Antimycobacterial Activity of Novel Imidazo[1,2-*a*]pyridine amide-cinnamamide Hybrids

Linhu Li, Zhuorong Li, Mingliang Liu, Weiyi Shen, Bin Wang, Huiyuan Guo and Yu Lu

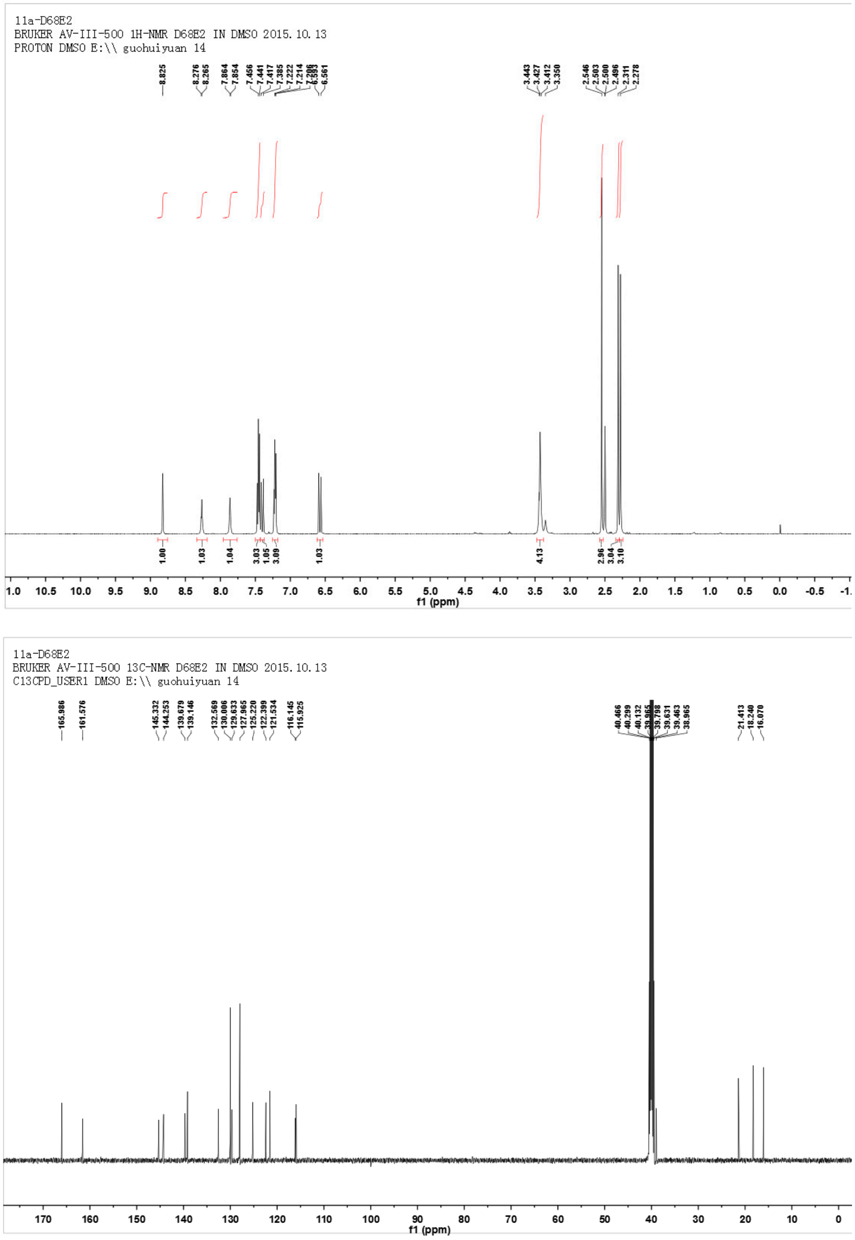

Figure S1. Copies of ^{1}H- and ^{13}C-NMR Spectra of **11a**.

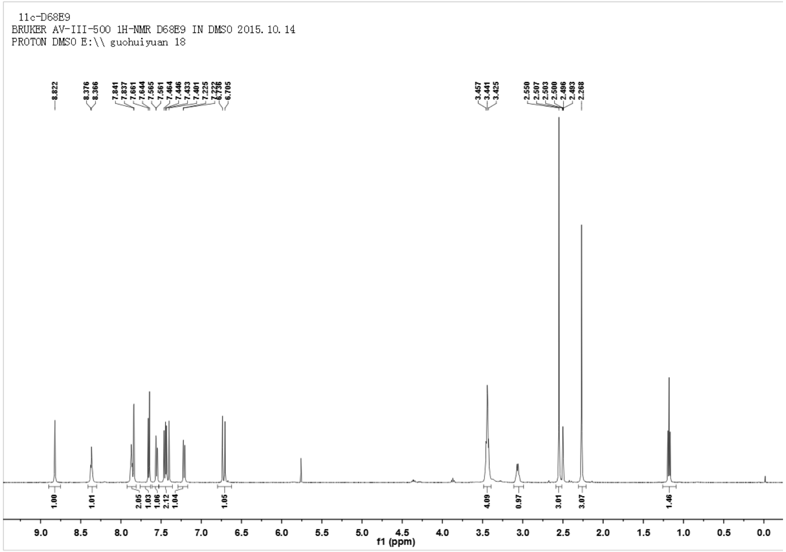

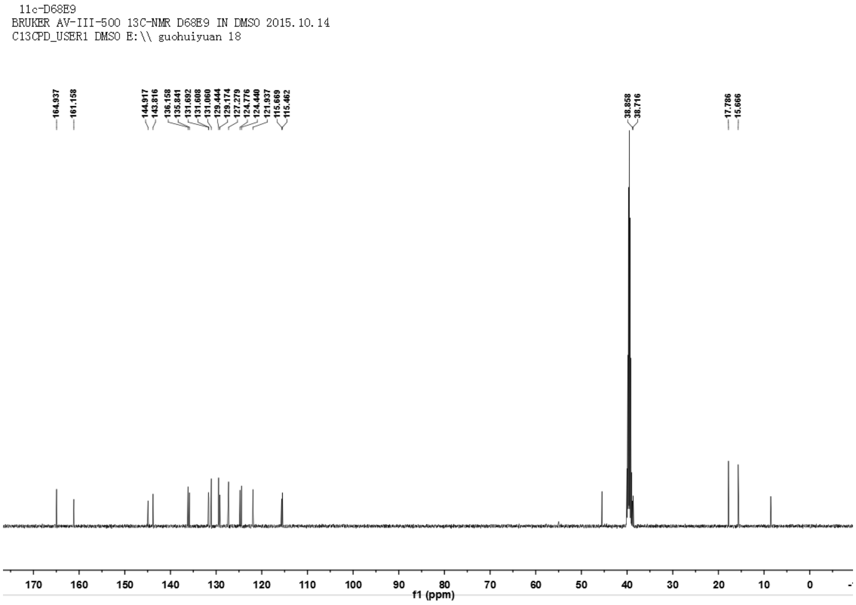

Figure S2. Copies of ^{1}H- and ^{13}C-NMR Spectra of **11c**.

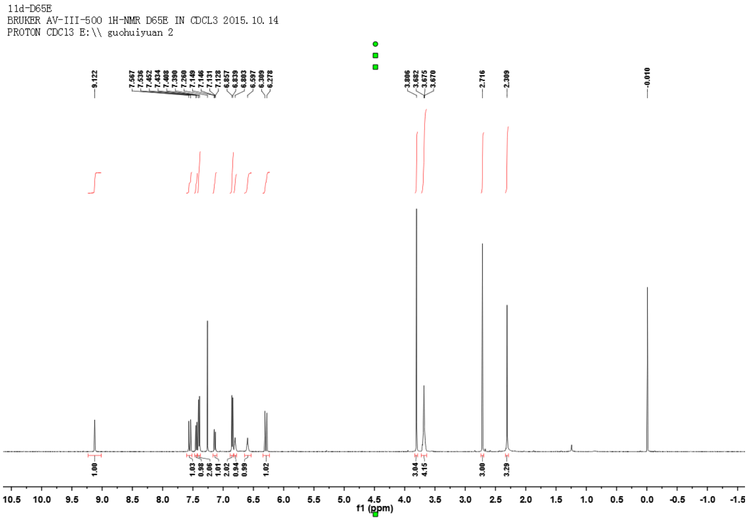

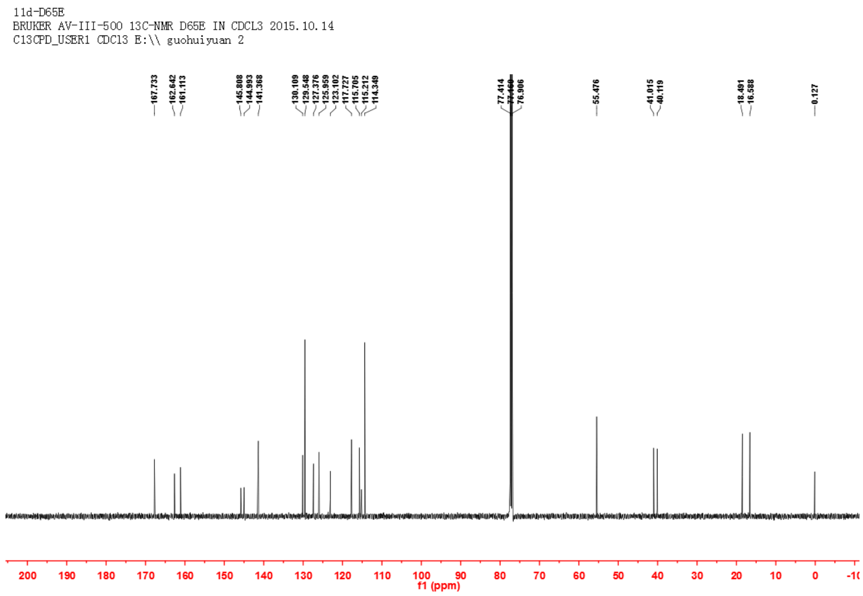

Figure S3. Copies of ^{1}H- and ^{13}C-NMR Spectra of **11d**.

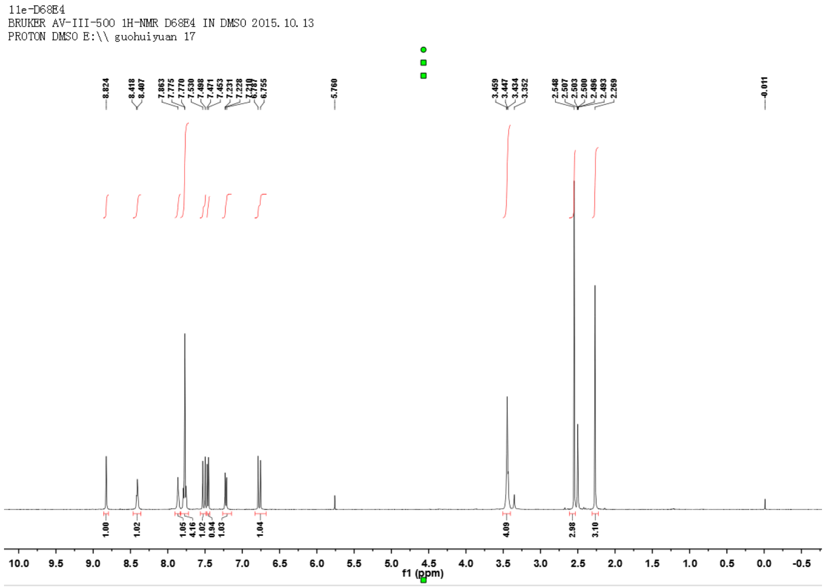

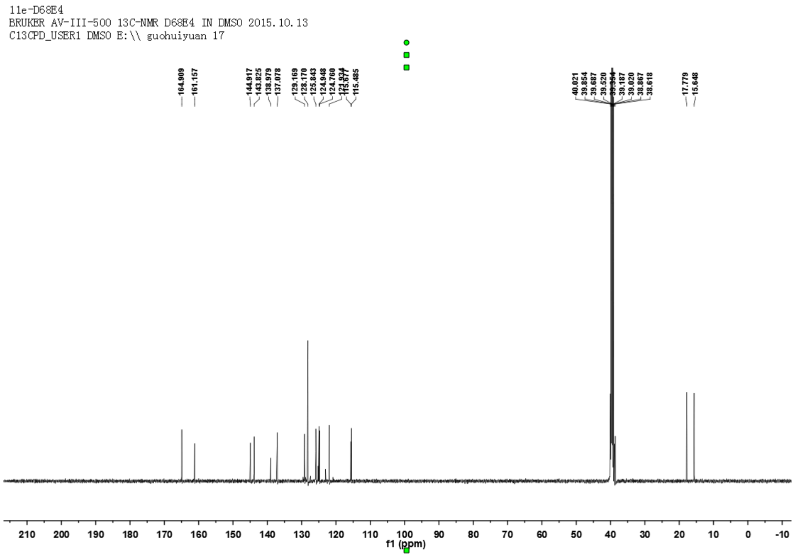

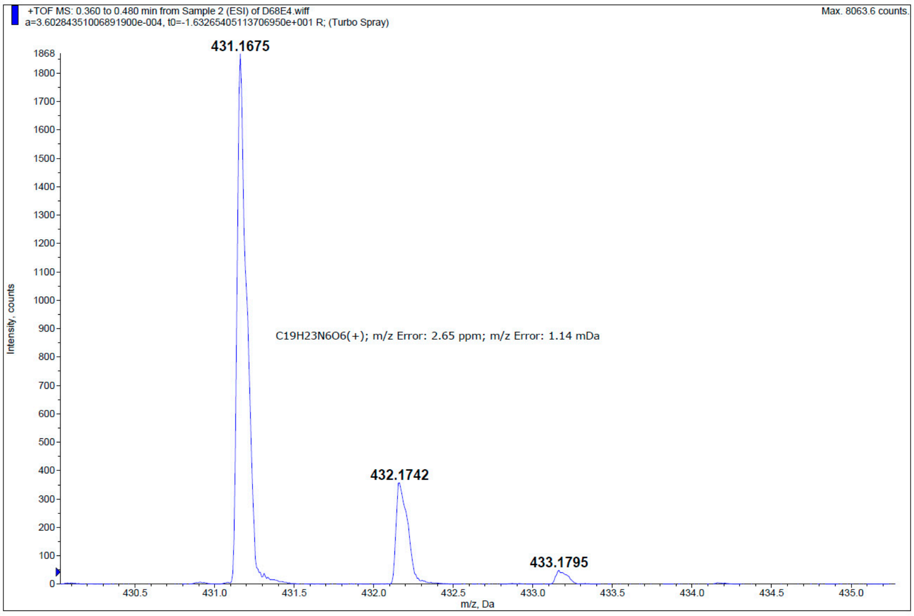

Figure S4. Copies of ^{1}H-, ^{13}C-NMR and HRMS Spectra of **11e**.

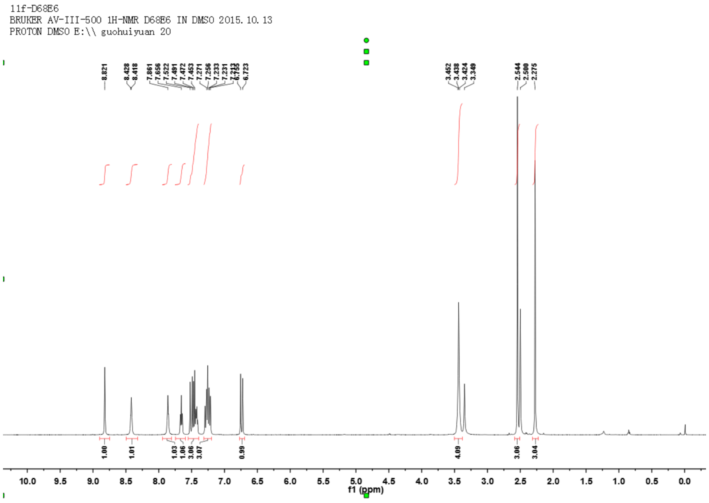

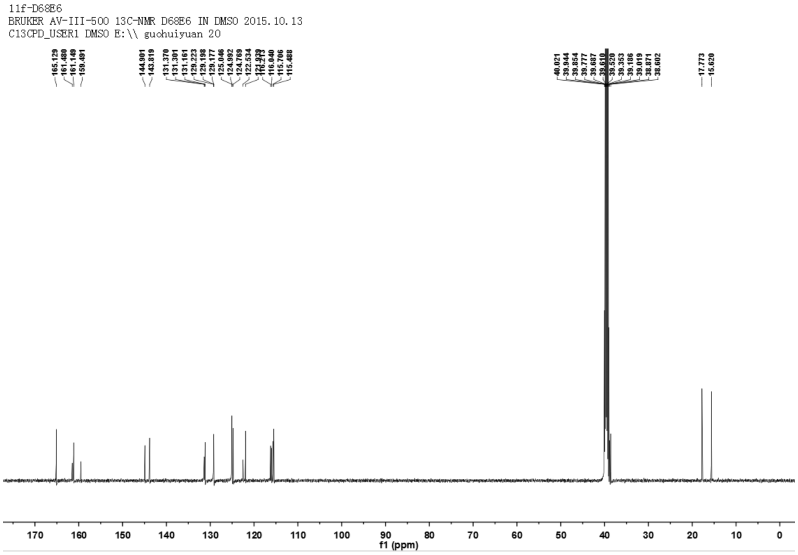

Figure S5. Copies of ^{1}H- and ^{13}C-NMR Spectra of **11f**.

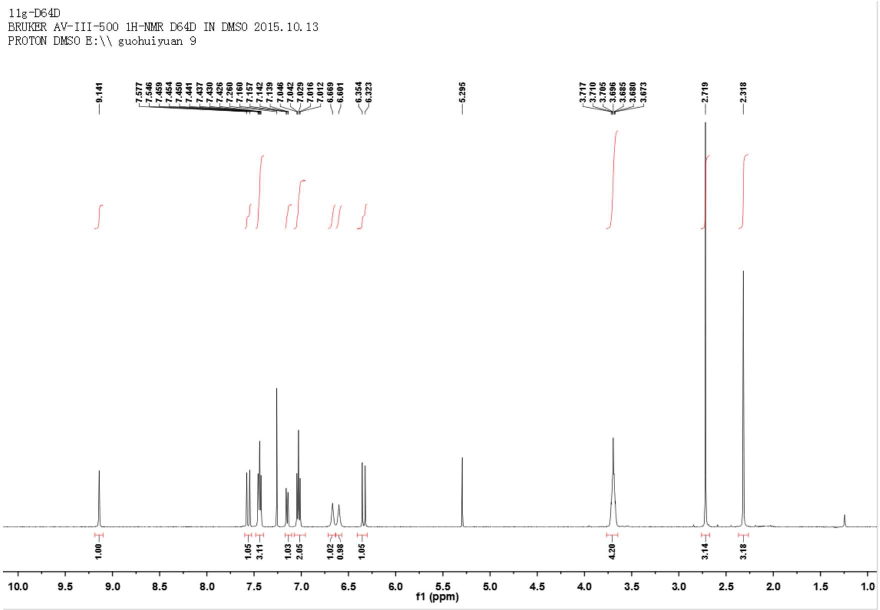

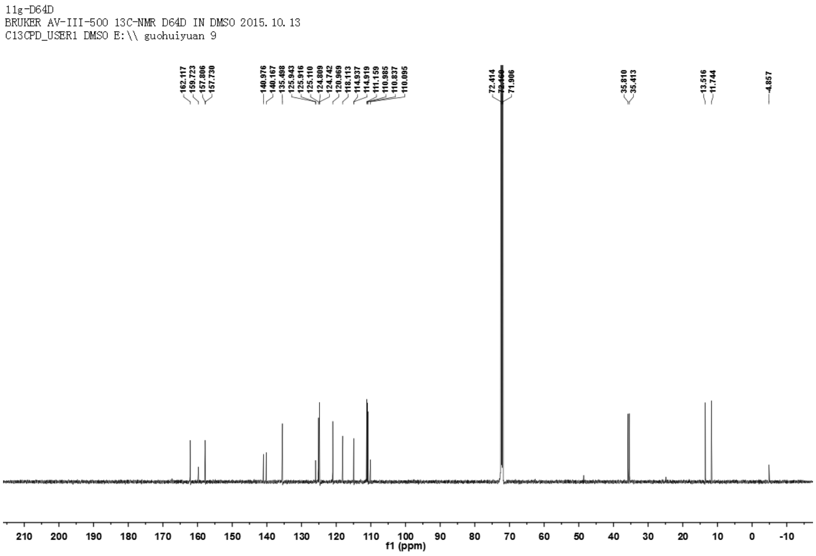

Figure S6. Copies of ^{1}H- and ^{13}C-NMR Spectra of **11g**.

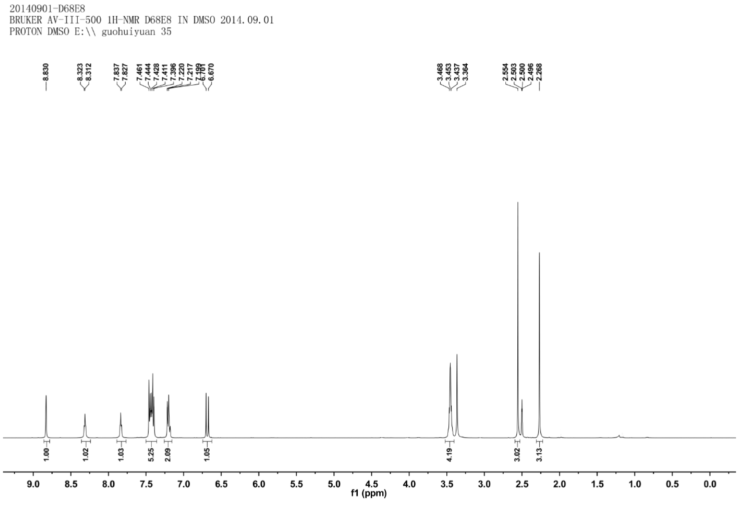

Figure 7. *Cont.*

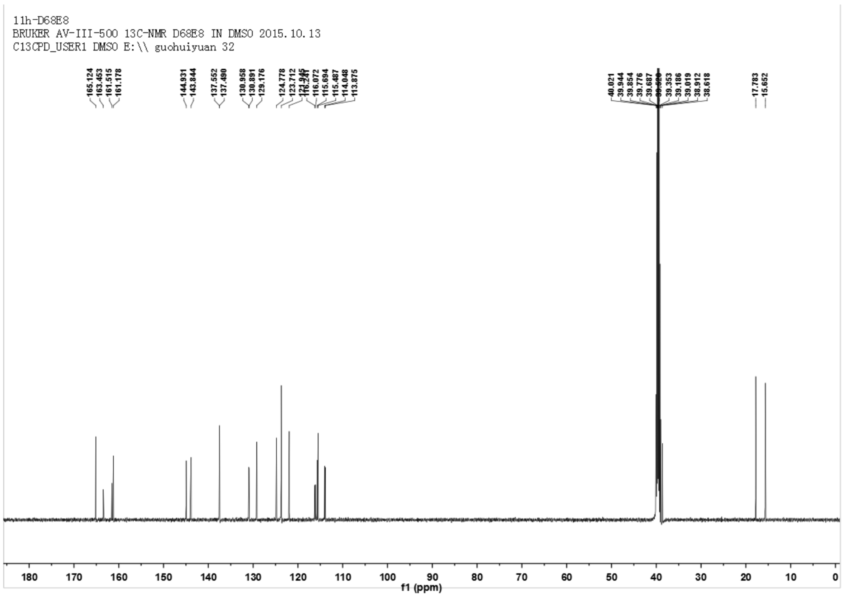

Figure S7. Copies of ^{1}H- and ^{13}C-NMR Spectra of **11h**.

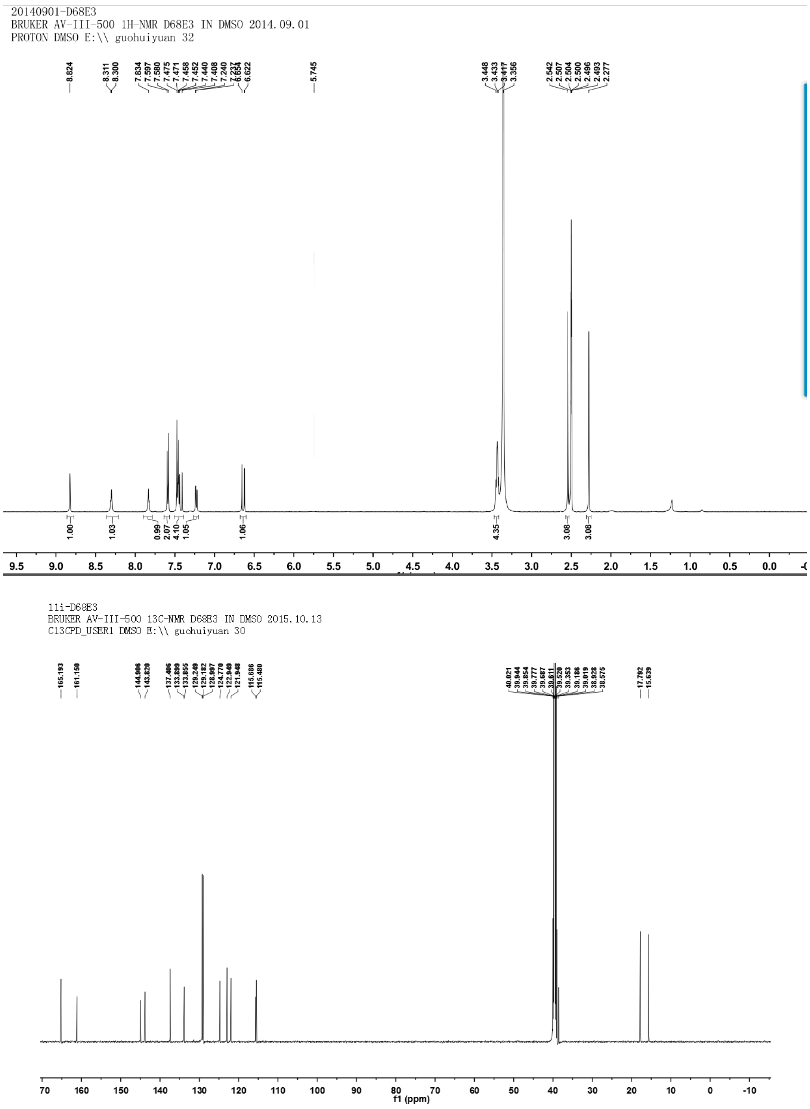

Figure S8. Copies of ^{1}H- and ^{13}C-NMR Spectra of **11i**.

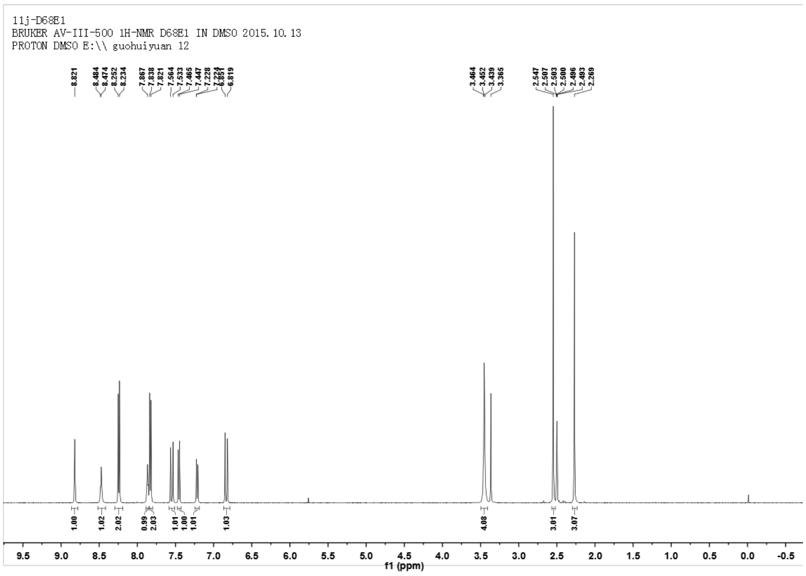

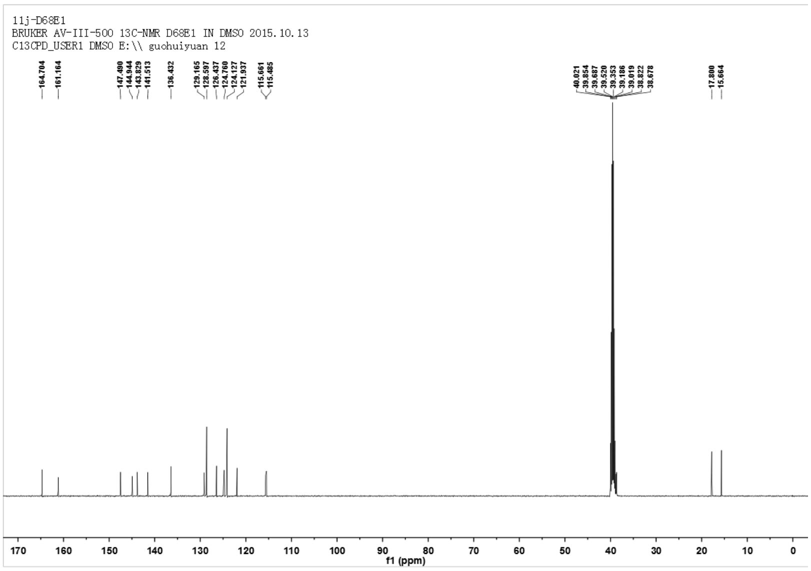

Figure S9. Copies of ^{1}H- and ^{13}C-NMR Spectra of **11j**.

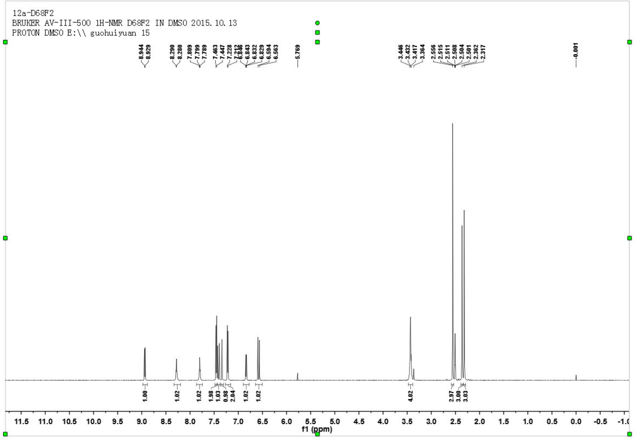

Figure 10. *Cont.*

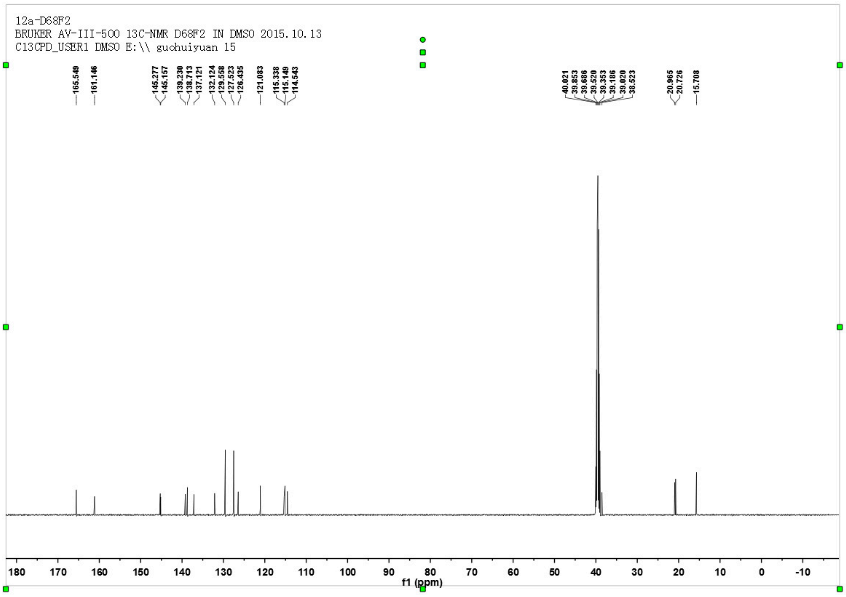

Figure S10. Copies of ^{1}H- and ^{13}C-NMR Spectra of **12a**.

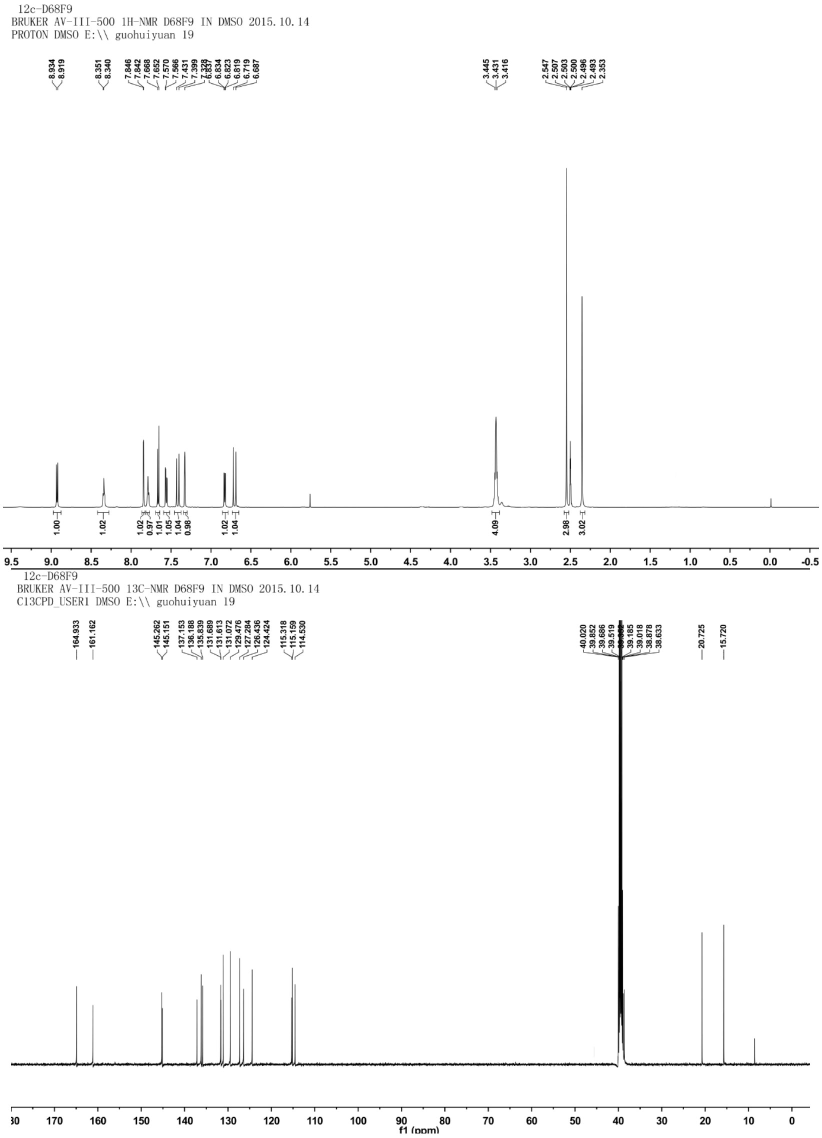

Figure S11. Copies of ^{1}H- and ^{13}C-NMR Spectra of **12c**.

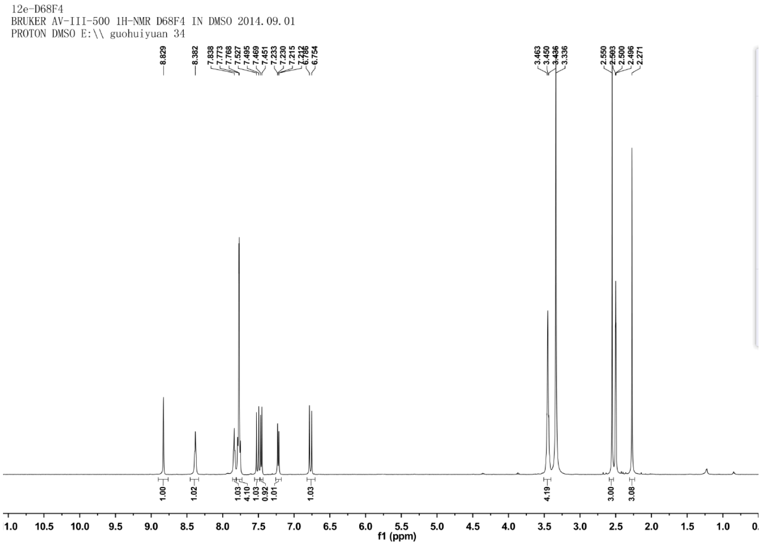

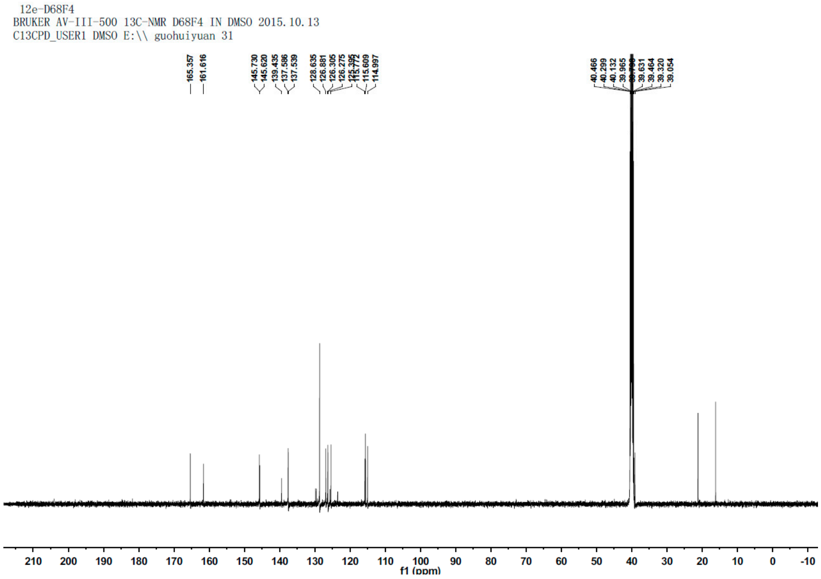

Figure S12. Copies of ^{1}H- and ^{13}C-NMR Spectra of **12e**.

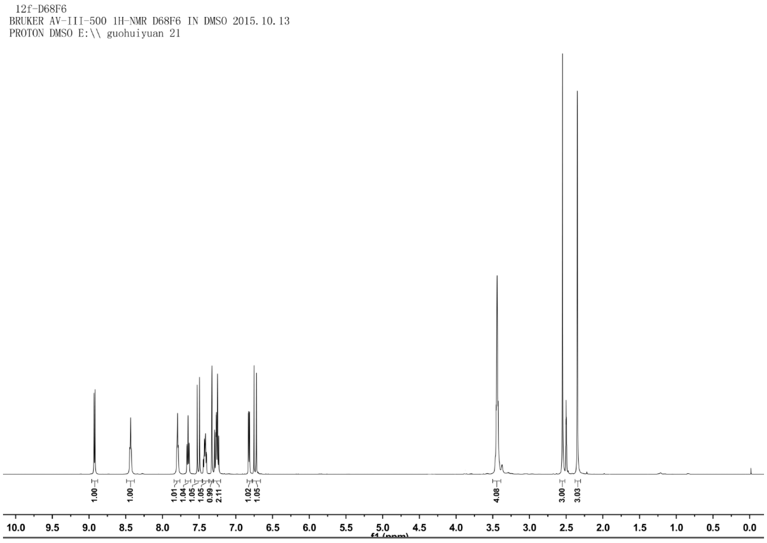

Figure 13. *Cont.*

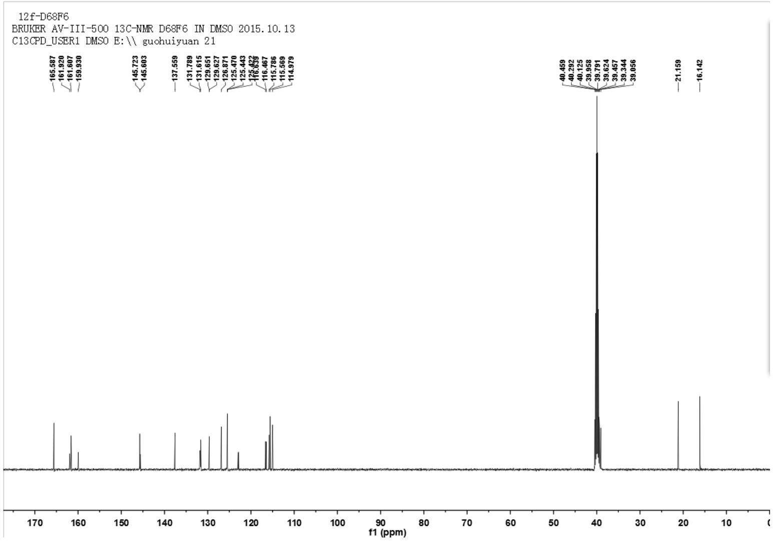

Figure S13. Copies of ^{1}H- and ^{13}C-NMR Spectra of **12f**.

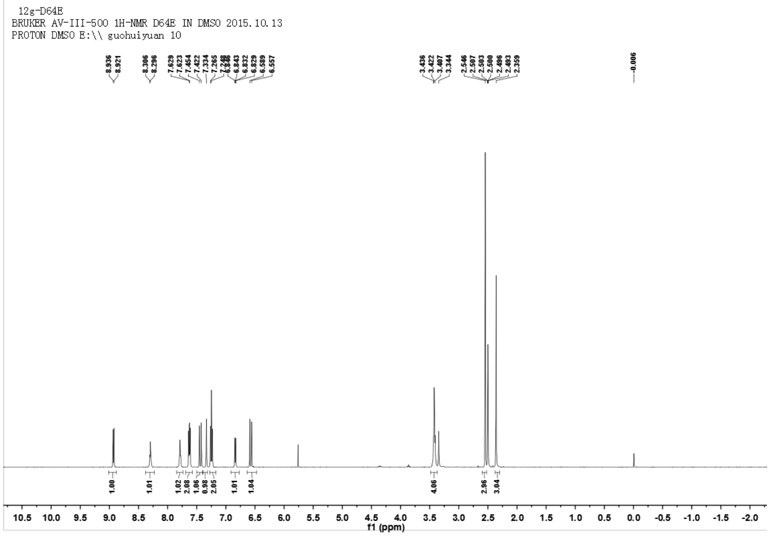

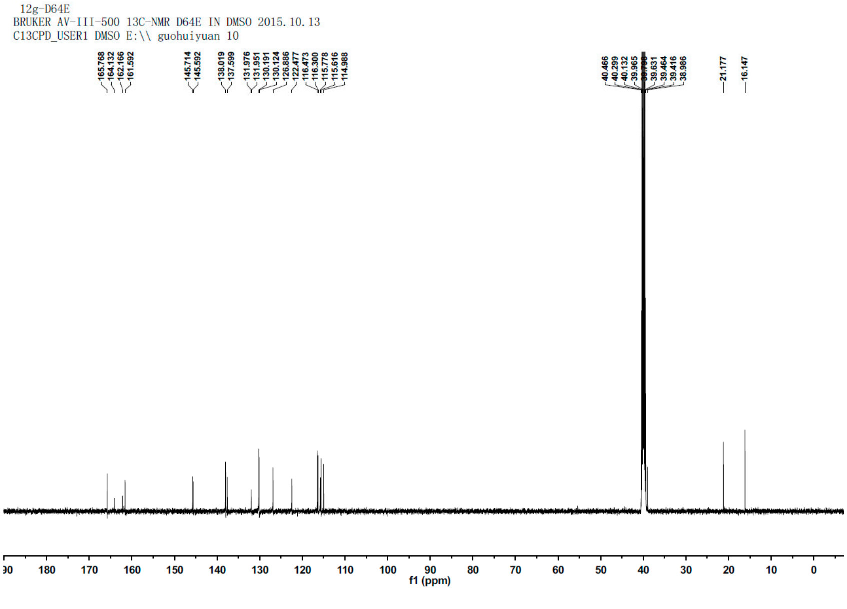

Figure S14. Copies of ^{1}H- and ^{13}C-NMR Spectra of **12g**.

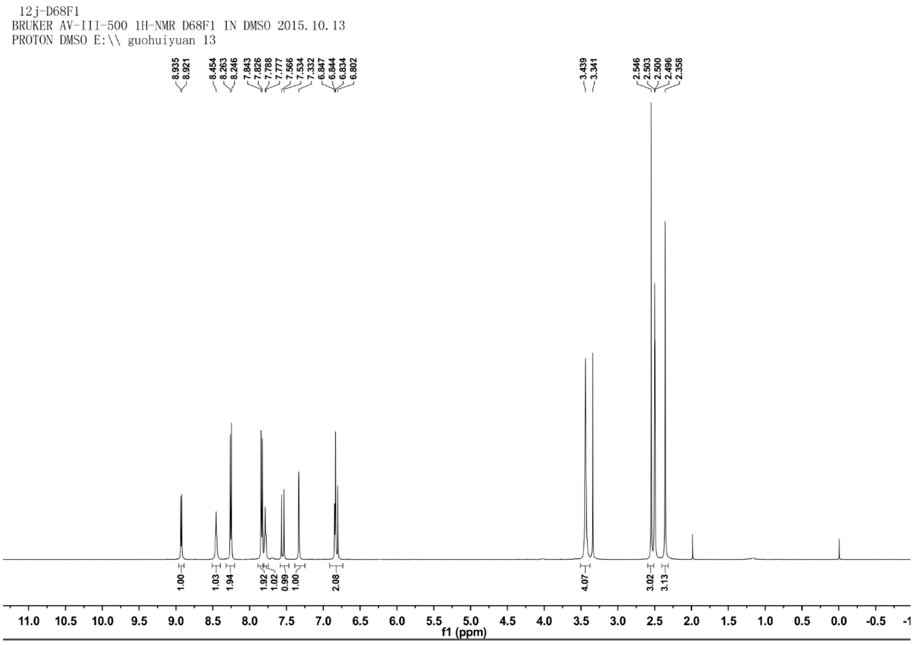

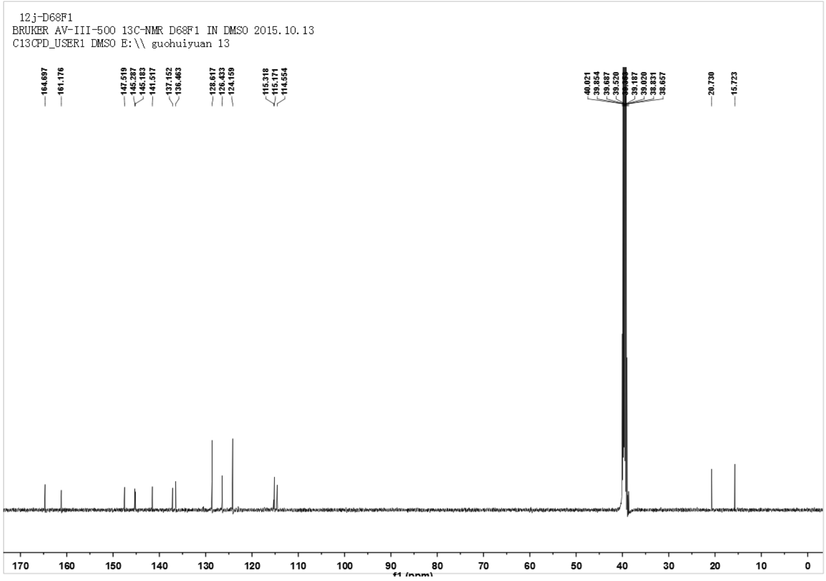

Figure S15. Copies of ^{1}H- and ^{13}C-NMR Spectra of **12j**.

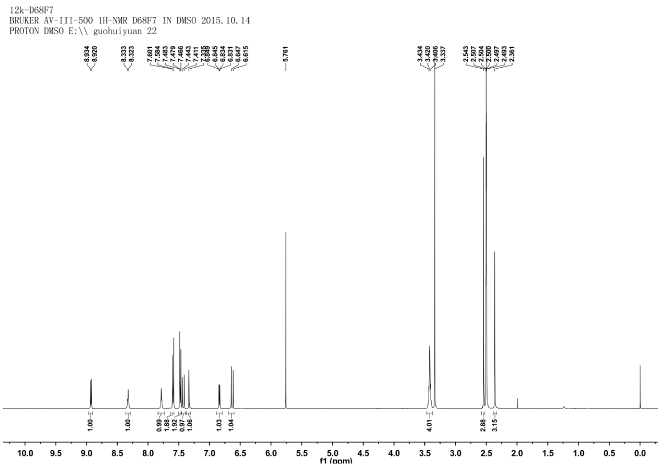

Figure 16. *Cont.*

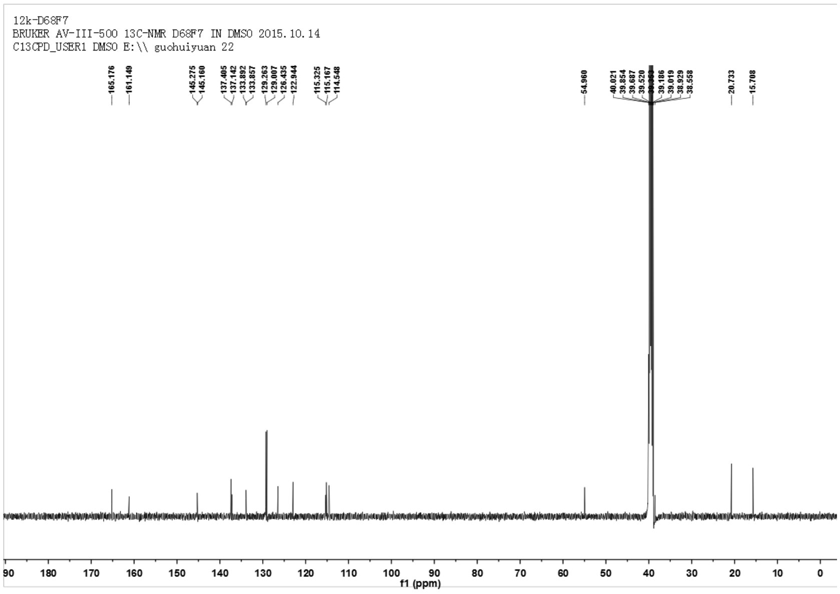

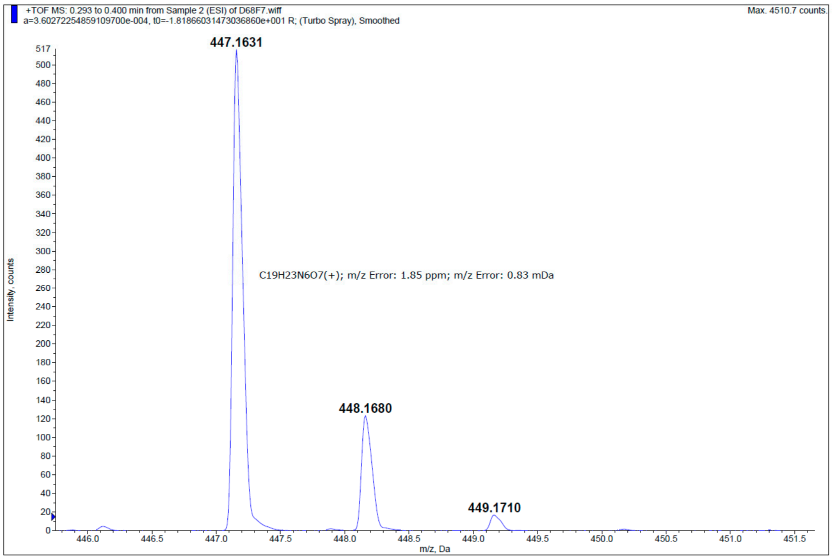

Figure S16. Copies of ^{1}H-, ^{13}C-NMR and HRMS Spectra of **12k**.

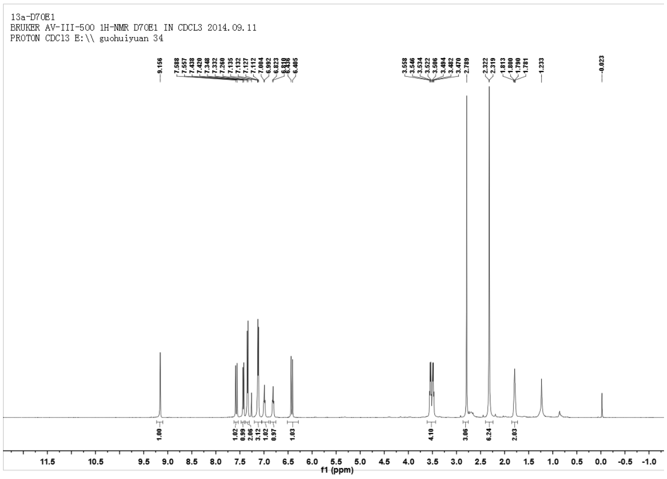

Figure 17. *Cont.*

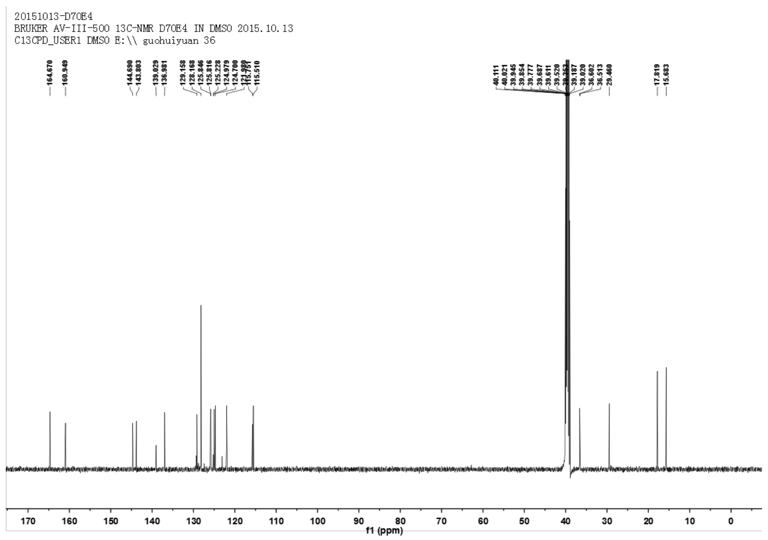

Figure S17. Copies of ^{1}H- and ^{13}C-NMR Spectra of **13a**.

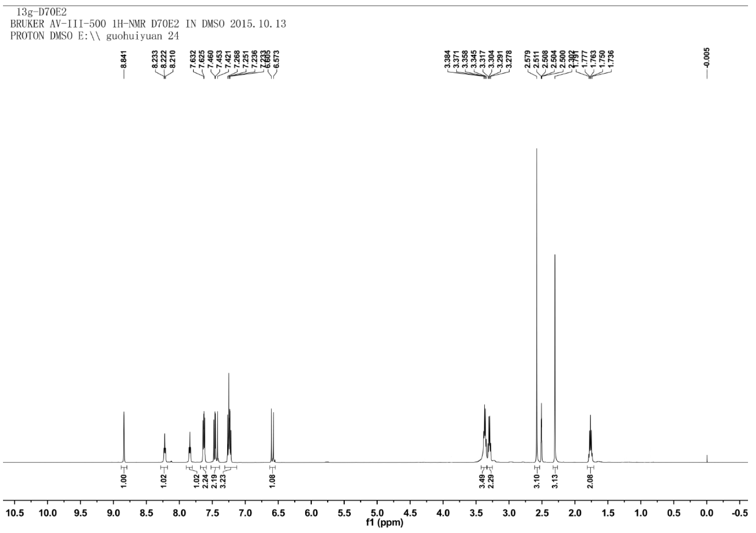

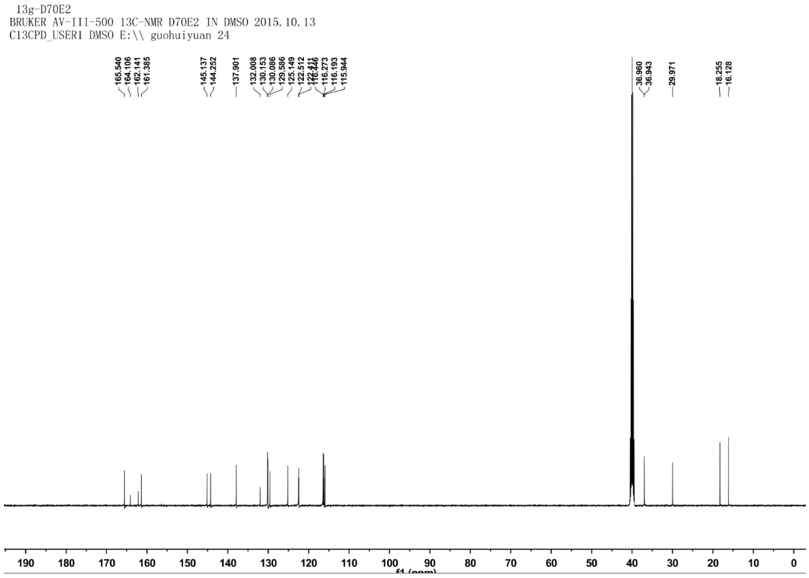

Figure S18. Copies of ^{1}H- and ^{13}C-NMR Spectra of **13g**.

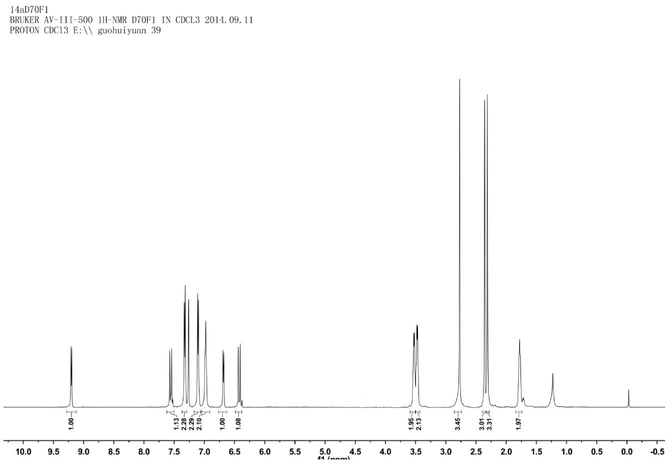

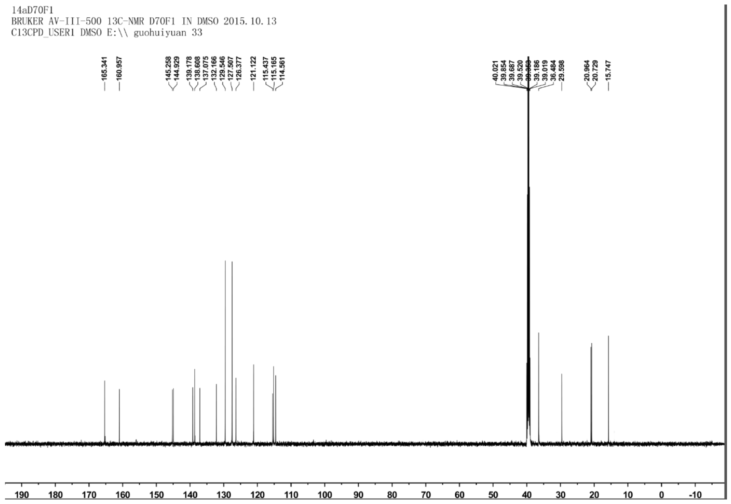

Figure S19. Copies of ^{1}H- and ^{13}C-NMR Spectra of **14a**.

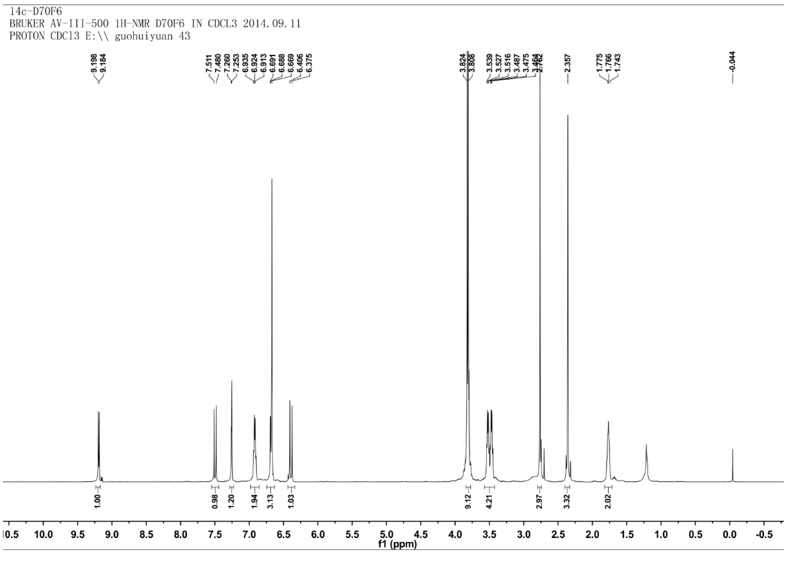

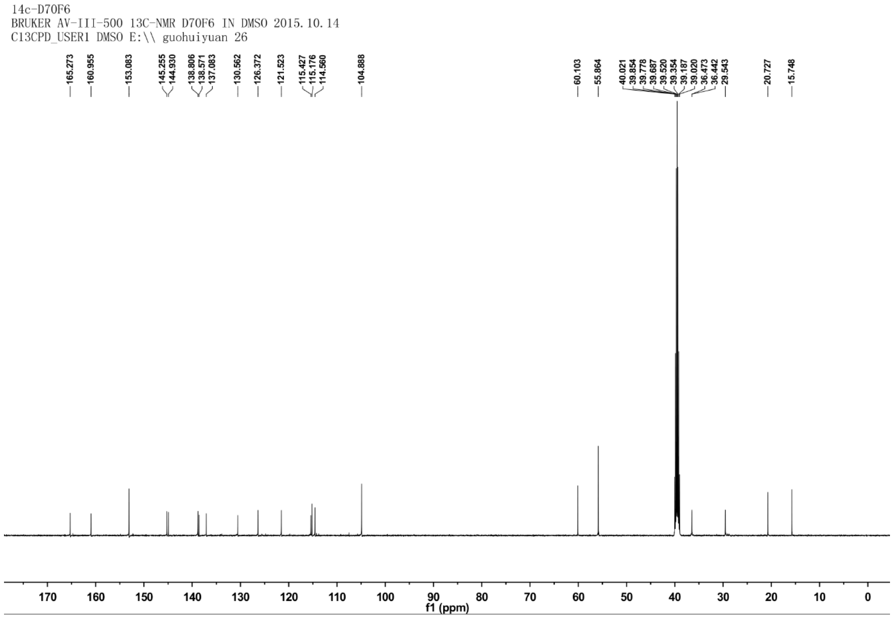

Figure S20. Copies of ¹H- and ¹³C-NMR Spectra of **14c**.

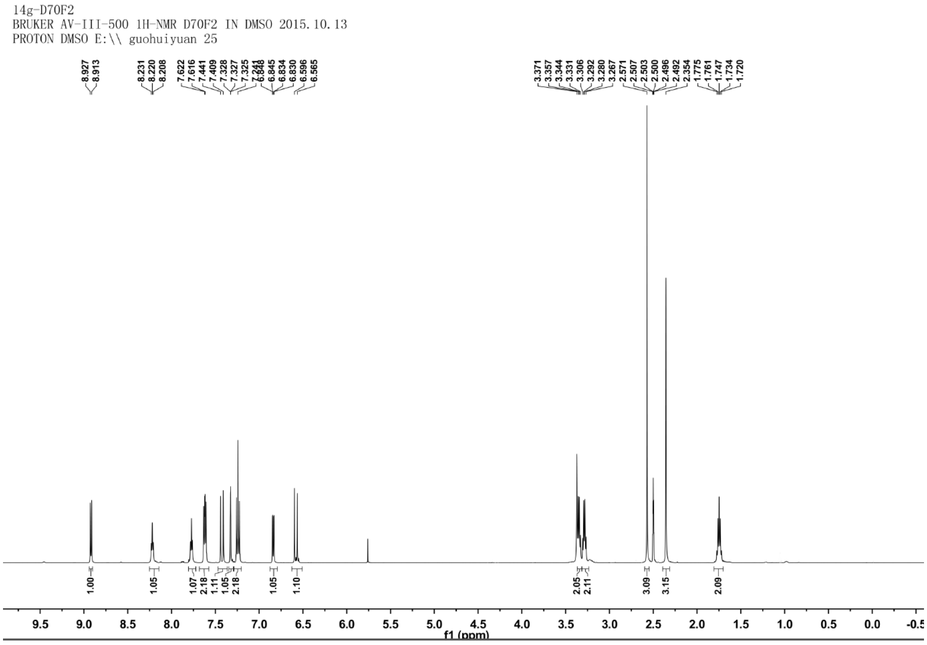

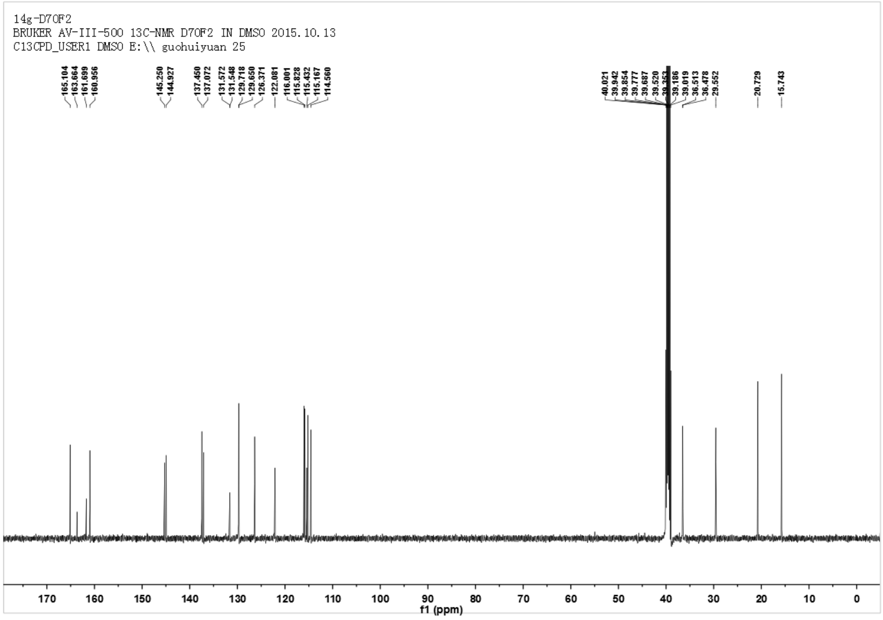

Figure S21. Copies of ^{1}H- and ^{13}C-NMR Spectra of **14g**.
